# Poor numerical performance of guppies tested in a Skinner box

**DOI:** 10.1038/s41598-020-73851-1

**Published:** 2020-10-07

**Authors:** Elia Gatto, Alberto Testolin, Angelo Bisazza, Marco Zorzi, Tyrone Lucon-Xiccato

**Affiliations:** 1 Department of General Psychology, University of Padova, Padua, Italy; 2 Department of Information Engineering, University of Padova, Padua, Italy; 3 Padova Neuroscience Center, University of Padova, Padua, Italy; 4 IRCCS San Camillo Hospital, Venice, Italy; 5 Department of Life Sciences and Biotechnology, University of Ferrara, Ferrara, Italy

**Keywords:** Behavioural methods, Animal behaviour, Cognitive neuroscience

## Abstract

We tested the hypothesis that part of the gap in numerical competence between fish and warm-blooded vertebrates might be related to the more efficient procedures (e.g. automated conditioning chambers) used to investigate the former and could be filled by adopting an adapted version of the Skinner box in fish. We trained guppies in a visual numerosity discrimination task, featuring two difficulty levels (3 vs. 5 and 3 vs. 4) and three conditions of congruency between numerical and non-numerical cues. Unexpectedly, guppies trained with the automated device showed a much worse performance compared to previous investigations employing more “ecological” procedures. Statistical analysis indicated that the guppies overall chose the correct stimulus more often than chance; however, their average accuracy did not exceed 60% correct responses. Learning measured as performance improvement over training was significant only for the stimuli with larger numerical difference. Additionally, the target numerosity was selected more often than chance level only for the set of stimuli in which area and number were fully congruent. Re-analysis of prior studies indicate that the gap between training with the Skinner box and with a naturalistic setting was present only for numerical discriminations, but not for colour and shape discriminations. We suggest that applying automated conditioning chambers to fish might increase cognitive load and therefore interfere with achievement of numerosity discriminations.

## Introduction

An understanding of the processes that underlie the main cognitive functions such as perception, learning, memory and attention, is historically due to studies carried out on animal species such as dogs, rats, pigeons and non-human primates^1^. Great impetus to the research in this field came from the introduction of automated devices that allow gathering considerable information in a short time and with minimal interference from the experimenter on the subjects^2^. Even today, much of the research takes place using computerized systems, such as Skinner-boxes, touchscreen training devices or systems for automatic tracking of behaviour^3^.

Over the past two decades, research has turned to investigating numerical cognition even in organisms that had not previously been considered, namely cold-blooded vertebrates and invertebrates (amphibians:^4^; fish:^5^; insects:^6^; molluscs:^7^). However, the research in these species does not usually make use of automated devices. This probably has various explanations, such as the often-small size of these animals and the fact that they sometimes live in a peculiar environment (e.g., aquatic habitats). Another important factor is that most training devices are based on food reinforcements; cold-blooded animals have low energy requirements and hence they eat much less and more infrequently^8^. Scarce use of automatic devices might thus limit research progress in these taxa as compared to mammals and birds^5^.

The disparity in methods also makes it more difficult to directly compare different taxa. For example, recent research has shown many fish species possess numerical capabilities^5^. Guppies, *Poecilia reticulata*, can be trained to discriminate, based on numerosity, up to 4 vs. 5 items^9^, to distinguish a specific numerosity, such as “4”, from other numerosities presented^10^, or to choose the 3rd or 5th object in a row of identical objects^11^. However, fish performance in numerical training is usually lower compared to warm-blooded species, which can learn more difficult discriminations (rhesus macaques, 7 vs. 8:^12^; pigeons, 6 vs. 7:^13^) or, in the case of chimpanzees, can order up to ten numerosities from the largest to the smallest^14^. Another crucial difference is that these latter species easily exceed accuracies of 90% correct responses^12,14^, while fish rarely reach a 75% accuracy. At least part of the observed gap between fish and warm-blooded species could be due to the fact that mammals and birds usually undergo several thousand trials with automated operant conditioning procedures to learn these tasks, while normally fish subjects undergo a few dozen trials and rarely exceed one hundred trials^9^. In line with this interpretation, recent work in the goldfish has suggested that if subjected to similar number of training trials as mammals and birds, fish too can reach very high accuracies (90% correct responses), at least in the easy numerosity discrimination range that has been used in this study (e.g. 0.33 to 0.67 ratios;^15^). Training fish requires however exceedingly long time, which makes the procedure unpractical and may expose animals to prolonged stress. For instance, in the study of DeLong et al.^15^, subjects received approximately 1500 trials and the training of four goldfish required more than 1 year.

We recently developed a Skinner box-like operant conditioning chamber for fish, equipped with a computerized system that tracks the movements of the subject and delivers small amounts of food upon a correct response^16^. Tested with this chamber, guppies can easily perform 80 reinforced trials per session and show excellent performance in colour discrimination, reaching a 90–95% accuracy in 2–3 sessions^16^. To test the hypothesis that part of the perceived gap between fish and homeotherms could be due to the use of automatic training procedures in the latter, we studied visual numerosity discrimination in guppies using the aforementioned Skinner box. The experiment consisted of a numerosity discrimination task with two different difficulty levels (3 vs. 5 and 3 vs. 4 items), using sets of stimuli controlled for non-numerical cues to obtain insight into the mechanism underlying quantity discrimination.

## Material and methods

### Experimental subjects and housing conditions

Subjects were adult guppies from an ornamental strain (“snakeskin cobra green”) breed at the Department of General Psychology of University of Padova. This population was the same as used in the recent studies conducted in our laboratory on Skinner-box training as well as on numerical abilities^9,16^. We trained 6 guppies, a sample size similar to that of prior studies on fish trained numerical abilities^9,15^. Two additional guppies participated to the pre-training phase of the experiment (see “Procedure”) but were not admitted to the test phase because they did not pass the learning criterion. Before the experiment, fish were maintained in mixed-sex groups of 20 individuals in large tanks (60 × 40 × 35 cm, 75 L). The maintenance tanks were provided with gravel bottom and natural vegetation. Biological and mechanical filters kept the water condition constant. Water temperature was kept at 27 ± 1 °C, and a fluorescent lamp provided illumination 12 h per day. Fish were fed three times per day with *Artemia salina* and commercial flakes (AquaTropical, Isola Vicentina, Italy).

### Automatic conditioning chamber

**Figure 1:**
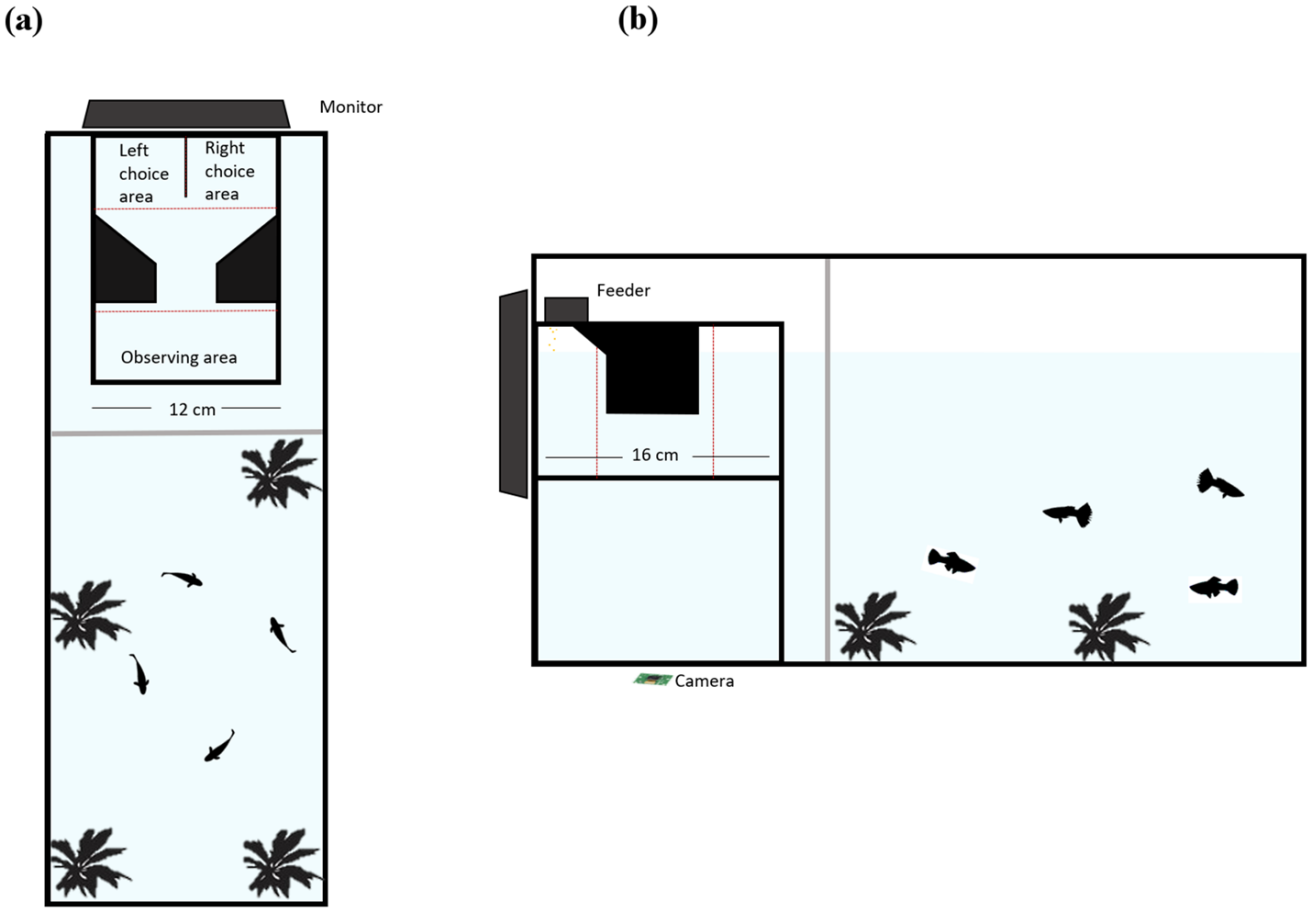
Top view (**a**) and side view (**b**) of the experimental tank with the housing compartment and the conditioning chamber. Red dotted lines indicate the boundaries of the areas used to track the subject in the conditioning chamber. The image was drawn by the authors with Microsoft PowerPoint^®^.

We used an automatic conditioning chamber similar to that described in detail elsewhere^16,17^ (Fig. 1). It consisted of a 15 × 12 × 10 cm tank made of semi‐transparent white plastic (thickness 0.3 cm). The illumination was provided by an 18-W fluorescent lamp positioned 150 cm above the conditioning chamber. A transparent bottom of the chamber allowed a module camera, positioned 12 cm below, to track the behaviour of each subject during the trials. The apparatus was internally subdivided into four sectors: a 4 × 12 cm observing area, a 7 × 4 cm V-shaped corridor, and two 4 × 6 cm choice areas. Each choice area presented a 6 × 5 cm window where an LCD computer monitor (Samsung S19C450) projected the stimuli. Subjects could equally see both stimuli from the observing area. The V-shape corridor leads subjects into the choice areas. A feeder, activated by a servomotor, was placed above the chamber, between the two choice areas (Fig. 1). The feeder was filled with decapsulated *A. salina* eggs, which were used as food reward. To reward the fish, the feeder expelled 80 μg eggs into the conditioning chamber. The projection of stimuli, the tracking of subject, and the activation of the feeder were controlled by a Raspberry Pi system (Raspberry Pi 3 Model B V1.2, 2015) running custom‐made Python software.

The conditioning chamber was inserted into a compartment of a 30-L glass experimental tank (20 × 50 × 30 cm; Fig. 1). The remaining compartment of the experimental tank (housing compartment) was similar to the maintenance tanks and housed two subjects (one male and one female) and two immature guppies as social companions. Subjects were individually moved from the housing compartment to the conditioning chamber before each experimental session. The two compartments of the tank were divided by a plastic wall with holes that permitted water exchange between the housing compartment and the conditioning chamber.

### Procedure

#### Pre-training phase

All experiments were conducted from September 2019 to April 2020. Initially, subjects underwent a pre-training phase in which they had to learn the general functioning of the conditioning chamber. One male and one female were transferred into the housing compartment of the experimental tank 1 week before the start of pre-training phase. During this week, the subjects were fed as in the maintenance conditions. The pre-training phase consisted of a series of 30-min sessions. Before each session, the experimenter netted the subject from the housing compartment and released it into the conditioning chamber. After 2 min of habituation, the session started and the computer presented a white background stimulus in each choice area. Then, as the subject entered the observing area, two identical stimuli (dark grey, 5 × 4-cm rectangle) were presented in each choice area. Once the subject entered one choice area, the reward was released from the feeder and the stimuli were substituted with the white background. To start a new trial, the subject had to move back into the observing area.

The maximum number of trials allowed per session was 80. The session ended when the subject obtained all the reinforcements or after 30 min. Two daily pre-training sessions, separated by a 2-h interval, were administered to each subject. The first daily session took place between 10:00 h and 12:00 h; the second daily session between 14:00 h and 16:00. To keep subjects in good health and to guarantee constant motivational level regardless of training’s success rate, at the end of the second daily session they were feed ad libitum with live *A. salina* naupli. Subjects were hence food deprived for approximately 16 h before the session of the following day.

During the sessions, the subject was left undisturbed in the conditioning chamber, with no human intervention. Accordingly, the subject had to spontaneously learn the general functioning of the chamber via exploratory behaviour, i.e. the food was released upon approaching the stimuli and a new trial was activated by swimming back to the observing area. In prior studies with this paradigm, we found that most fish were highly successful in learning the functioning of the conditioning chamber^16,17^. We ensured that this occurred in the present study before admitting the subjects to the testing phase. In particular, we used a criterion based on number of trials performed and direct observation via the camera. Once a subject consumed at least 30 reinforcements per session in two sessions, it was admitted to the training phase. Two subjects did not reach this criterion after 12 pre-training sessions. We considered that these two subjects failed in learning the functioning of the conditioning chamber; therefore, we did not admit them to the numerosity discrimination phase.

#### Numerosity discrimination phase

In this phase, we evaluated subjects’ discrimination performance. The numerosity discrimination phase consisted in a series of 60 min daily-sessions administered to each subject. Sessions were performed between 10:00 h to 14:00. The first trial of each session started when the subject entered the observing area and the monitor projected a randomized pair of numerical stimuli in the window of each choice area (see details on the stimuli below). All subjects were trained to choose the larger stimulus. This was done to allow comparison with recent studies in the same species^9^ and because guppies and other fish species show no difference in accuracy when trained with the smaller or the larger numerosity as positive^10,18^. The larger stimulus was randomly presented in correspondence of the right or left choice area in each trial, with the only constraint of not using the same position for more than 3 consecutive trials. Once the subject entered the choice area corresponding to the larger stimulus, the system recorded a correct choice and activated the release of the food reward. Afterwards, the monitor projected a white background, and a new trial started as soon as the fish moved to the observing area. If the subject entered the choice area corresponding to the smaller stimulus, a black background was projected. The procedure allowed fish to correct their incorrect choices: the same pair of stimuli in the same left–right position was repeatedly presented until the subject made a correct choice (correction trials). The interval between an incorrect choice and the following correction trial was 10 s. This procedure permitted to uniform the number of reinforcements obtained by each subject.

We set the maximum number of reinforcements that subjects could obtain per session at 80^16,17^. The training session ended automatically when the subject obtained 80 reinforcements or when the time (60 min) expired. We administered a second 60-min training session in the same day if a subject obtained less than 40 reinforcements in the first session. As a measure of accuracy, we considered only the first choice for each stimulus presented (i.e., correction trials were not considered). Each subject performed a total of 500 trials, with the exception of one subject that stopped to feed during the experimental sessions, although we did not detect evident signs of distress or disease. We therefore analysed the data of this subject only until the last trial in which it was observed to consume the food (450 trials overall).

#### Numerical stimuli

**Figure 2:**
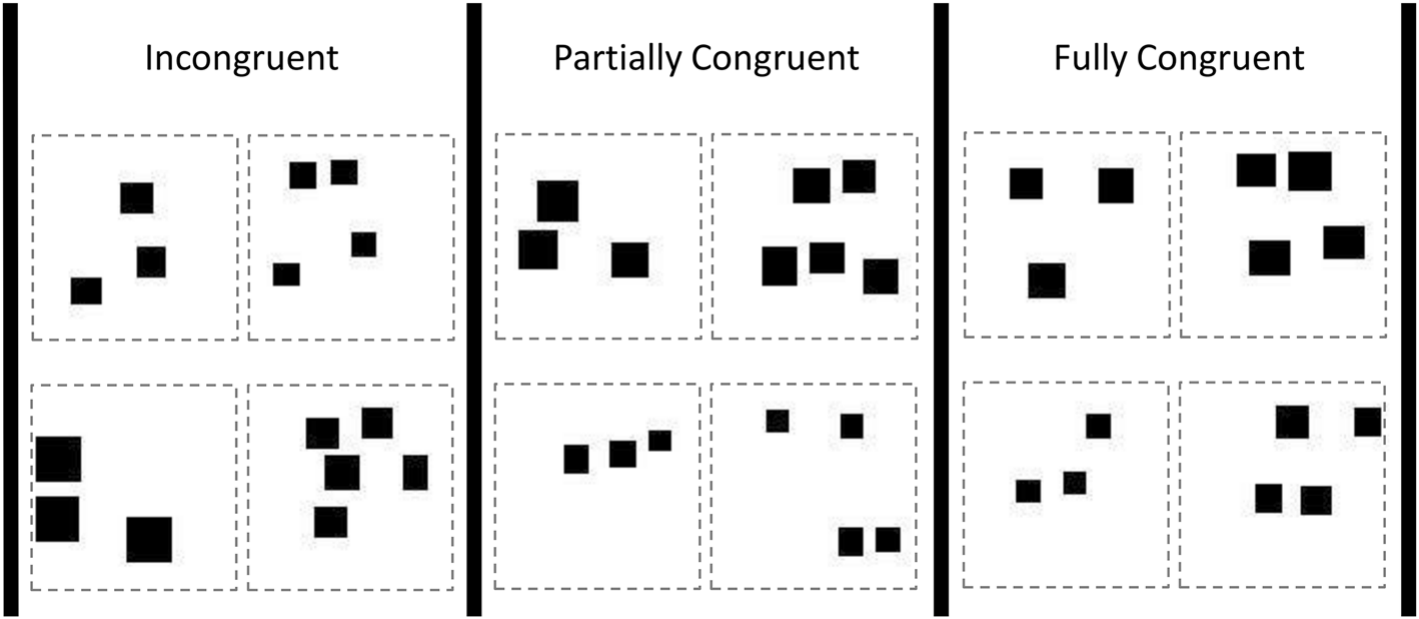
Samples of stimulus pairs used in the numerosity discrimination training, grouped according to different conditions of congruency between numerosity information and continuous cues. The image was drawn by the authors with MATLAB.

Stimuli consisted in bitmap images containing a variable number of black rectangular items, randomly placed into a white background of size 96 × 96 pixels (Fig. 2). They were automatically generated with a MATLAB script implemented for previous research^19^. Size of the items ranged from 0.19 × 0.19 cm (visual angle: 1° 27′ 01″) to 0.66 × 0.66 cm (visual angle: 5° 2′ 33′). It was demonstrated as guppies respond to stimuli with visual angle similar to and even smaller than that used in our study (1° 15′ 63″)^20^. Moreover, prior studies in this and other species often used this type of stimuli to investigate numerical discrimination^18,21,22^. Stimuli were combined into pairs to produce trials with different numerical ratio, either 0.6 (3 vs. 5) or 0.75 (3 vs. 4). According to prior research, guppies easily perform numerical discriminations with 0.6 ratio but they acquire discriminations with 0.75 ratio only with certain experimental paradigms^9,21,23,24^. Therefore, our choice of numerical ratios was well suited for investigating the efficiency the conditioning chamber to study guppies’ numerical abilities. In detail, if guppies succeeded in both the 0.6 and the challenging 0.75 numerical discriminations, we would conclude that the conditioning chamber improves numerical discrimination learning. Conversely, if guppies succeeded only with the easier ratio (0.6), we would conclude that the conditioning chamber allows to study learning but does not provide increased learning efficiency in the context of numerical discrimination. Besides manipulating numerosity, we also manipulated other visual features to investigate the impact of non-numerical magnitudes in discrimination performance (see^25^). In the “incongruent” condition, cumulative area was equated in the two stimuli, implying that individual item size was incongruent with number (i.e., stimuli with larger numbers had smaller items). In the “partially congruent” condition, average individual item size was equated in the two stimuli, implying that cumulative area was congruent with numerosity (i.e., stimuli containing more items also had a larger number of black pixels). Finally, in the “fully congruent” condition both cumulative area and item size were congruent with number.

### Statistical analyses

We performed the statistical analysis in RStudio version 1.1.383. Descriptive statistics are reported as mean ± SD. First, we assessed whether fish showed evidence of learning the task. For this scope, we analysed performance at the group level by comparing the proportion of correct choices of each subject in the entire experiment to chance level performance (i.e., a proportion of 0.5) using a one-sample *t* test. Then, we evaluated the individual performance of each subject in the numerical task using a binomial test on the number of correct and incorrect choices. In addition, to deal with our large dataset (approx. 3000 datapoints) characterised by repeated observations per each subject and binomial distribution (number of correct and incorrect choices), we run a generalized linear mixed-effects model with logit link function and binomial error distribution (GLMM, “glmer” function from the “lme4” R package) to assess learning as an accuracy increase over training. Due to the different number of reinforcements per daily session, we grouped trials in 10 blocks of 50 trials each. We fitted the model with block number as fixed effect, and subject as random effect. This model was also fitted with congruency condition (factor with three levels: incongruent condition; partially-congruent condition; fully-congruent condition), and numerical ratio (factor with 2 levels: 3 vs. 5 and 3 vs. 4) as fixed effects, allowing use to assess the effect of these factors on fish performance. We evaluated the effect of the parameters using ‘Anova’ function of the ‘car’ R package. When necessary, significant main effects of the model were analysed with Tukey post-hoc test. Significant interactions involving block were analysed by running additional models on the dataset split according to the levels of the factor involved. We further analysed significant effects and interactions with post-hoc one-sample *t* tests (*P* values were corrected for multiple comparisons with the Bonferroni method) on the proportion of correct responses of data split according to the levels of the factor of interest.

### Comparison with naturalistic training setting

To provide an assessment of guppies’ performance in our Skinner box relative to other training methods, we compiled a dataset including data from three prior studies performed with subjects of the same strain as our guppies^9,16,26,27^. Lucon-Xiccato and colleagues^16^ tested 8 guppies with the Skinner box, 4 subjects in a colour discrimination (red vs. green) and 4 subjects in a shape discrimination (black triangle vs. black circle). As a part of an investigation on sex differences in cognition^26,27^, 28 guppies were tested in a colour discrimination (red vs. yellow) and 20 guppies in two shape discriminations (black triangle vs. black square and horizontal bar vs. ‘S’ shape). These studies used a naturalistic setting in which fish had to dislodge the disc with the correct colour or shape to extract a food reward concealed underneath. Bisazza and colleagues^9^ tested 6 guppies in a numerical discrimination with the naturalistic setting; here, groups of discs with different numerosity were presented (2 vs. 3, 3 vs. 4, and 4 vs. 5) and food was hidden only under discs of the group with the larger numerosity. All the subjects were naïve and only tested in one experiment; accordingly, data of the various experiments were independent. As dependent variable, we used the accuracy of each guppy calculated as number of correct responses/number of trials. Therefore, the final data set consisted of accuracy of individual guppies in three types of discrimination (colour, shape and numerical), each one assessed with two methods (Skinner box and naturalistic setting). To analyse the data, we performed ANOVA fitted with training method (factor with two levels: Skinner box; naturalistic setting) and type of discrimination (factor with three levels: colour; shape; numerical). Data were rank transformed to deal with different distributions of the various experiments. In addition, we run post-hoc Wilcoxon tests to investigate the meaning of a significant interaction in the ANOVA.

### Ethical approval

The experiment of this study was conducted in accordance with the law of the country in which it was performed (Italy, D.L. 4 Marzo 2014, n. 26) and was approved by the Ethical Committee of University of Padova (protocol n. 196527/2016). After the experiment, all subjects were released in maintenance tanks.

## Results

### Overall performance

Group-level analysis showed that fish chose the correct stimulus in 56.2 ± 1.93% of trials, an accuracy that was significantly greater than chance level (one-sample *t* test: *t*_5_ = 7.849, *P* < 0.001). Moreover, the GLMM revealed that subjects’ accuracy significantly increased over training block ***(χ^2^_1_ = 5.092, *P* = 0.024). Individual-level analysis showed that 4 out 6 guppies chose the correct stimulus more often than expected by chance (Table 1).

**Table 1:** Performance of individual guppies in the numerosity discrimination task. For each individual, percentage correct responses (mean ± SD), number of correct responses/number of incorrect responses and *P* value calculated with the binomial test (one degree of freedom) are reported. Bold indicates subjects that chose the correct stimulus more often than chance.

	Subject 1	Subject 2	Subject 3	Subject 4	Subject 5	Subject 6
Performance	**58.80 ± 7.67%,** **294/500,** **P < 0.001**	54.00 ± 4.58%, 243/450, *P* = 0.100	**57.40 ± 7.83%,** **287/500,** **P = 0.001**	**55.20 ± 9.00%,** **276/500,** **P = 0.022**	**57.40 ± 4.12%,** **287/500,** **P = 0.001**	54.40 ± 6.72%, 272/500, *P* = 0.054

### Effect of ratio

**Figure 3:**
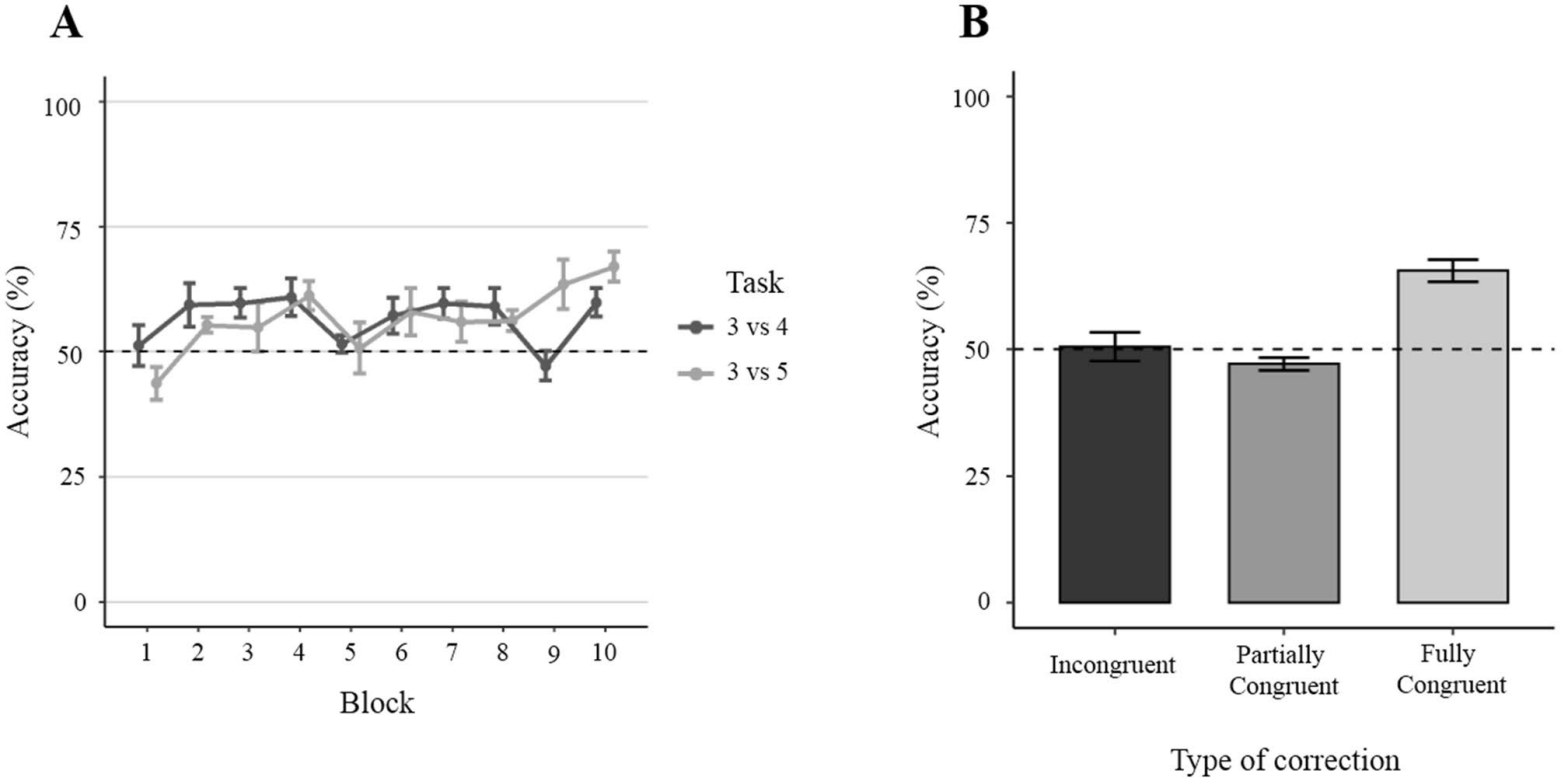
(**A**) Accuracy per block (50 choices) divided for 3 vs. 5 (light grey) and 3 vs. 4 (dark grey) numerosity discrimination. (**B**) Accuracy as a function of congruency condition. Data points represent mean ± SEM. Dotted lines represent chance performance (50% correct responses). The image was drawn by the authors with R Studio.

The GLMM revealed no significant main effect of ratio (*χ*^2^_1_ = 2.336, *P* = 0.126). However, there was a significant interaction between numerical ratio and block (*χ*^2^_1_ = 7.594, *P* = 0.006; Fig. 3A). The post-hoc analysis separated by numerical ratio indicated the cause of the interaction: subjects increased their accuracy over blocks in the 3 vs. 5 discrimination (*χ*^2^_1_ = 12.227, *P* < 0.001) but not in 3 vs. 4 discrimination (*χ*^2^_1_ = 0.803, *P* = 0.803). In support, analysis with one-sample *t* test showed that in the 3 vs. 5 discrimination subjects’ accuracy significantly differed from chance level in the last block (67.03 ± 6.32%, *P* Bonferroni adjusted = 0.038); a similar trend was found for the ninth block (63.46 ± 7.17%, *P* adjusted = 0.058). Conversely, in the 3 vs. 4 discrimination subjects’ accuracy did not significantly differ from chance level in any of the last two blocks (ninth block: 47.17 ± 12.22%, *P* adjusted = 1.000; tenth block: 59.85 ± 6.72%, P adjust = 0.306).

### Effect of congruency condition

The GLMM revealed that subjects’ accuracy significantly differed among the three congruency conditions (*χ*^2^_2_ = 58.397, *P* < 0.001). Tukey post-hoc test found that the accuracy of subjects was higher in the fully-congruent condition compared to the partially-congruent condition (*P* < 0.001) and the incongruent condition (*P* < 0.001), but no difference was found between the two latter conditions (*P* = 0.471). An additional post-hoc analysis with *t* test showed that subjects significantly chose the correct stimulus when continuous cues of the stimuli were fully congruent with numerosity (*t*_5_ = 7.061, *P* adjusted = 0.003; Fig. 3B). Conversely, subject’s accuracy did not differ from chance level when size was incongruent with number (*t*_5_ = 0.185, *P* adjusted > 0.999; Fig. 3B) and when only cumulative area was congruent with number (partially-congruent condition: *t*_5_ = 2.313, *P* adjusted = 0.206; Fig. 3B). Finally, the GLMM revealed no other significant interactions (all *P* values > 0.6).

### Comparison with naturalistic training setting

**Figure 4:**
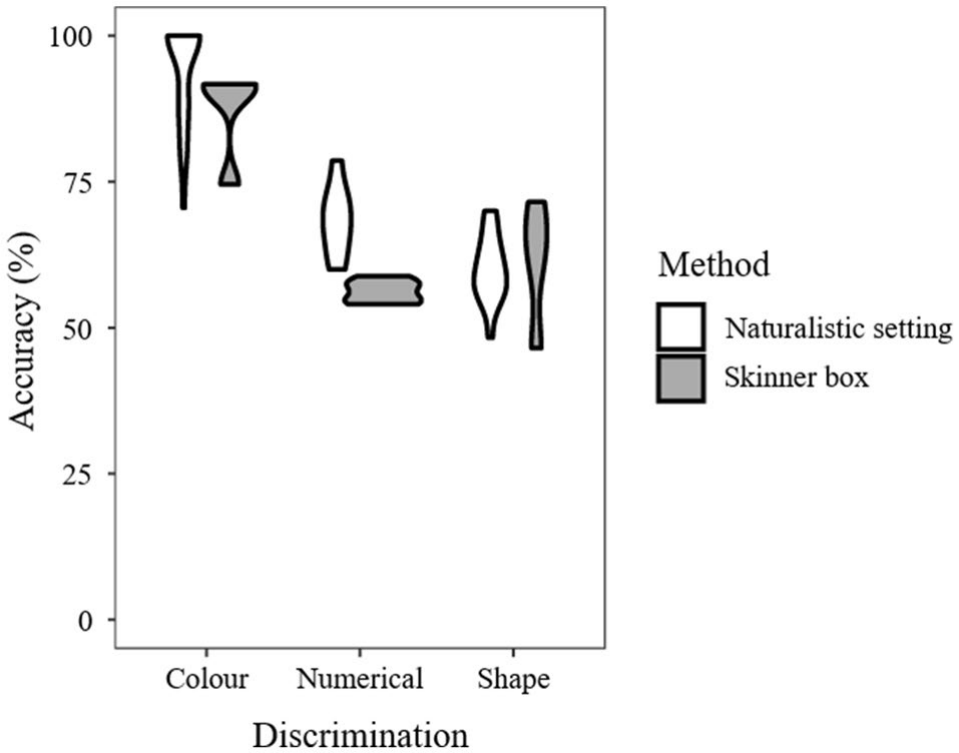
Violin plot of guppies’ accuracy in three types of visual discrimination (colour, shape and numerical) assessed with two training methods (Skinner box and a naturalistic setting in which guppies have to dislodge the correct object to find the reward underneath). Data were obtained from: the present study and four prior studies on the same fish species^9,16,26,27^. The image was drawn by the authors with R Studio.

The analysis on the pooled data of prior studies found a significant effect of method (*F*_1,64_ = 8.692, *P* = 0.004) and discrimination (*F*_2,64_ = 127.665, *P* < 0.001). However, there was a significant interaction between these two terms (*F*_2,64_ = 6.575, *P* = 0.003; Fig. 4). Post-hoc analysis revealed that guppies’ performance with the two methods was similar for colour (*W* = 81, *P* = 0.123) and shape discriminations (*W* = 31, *P* = 0.510). However, for numerical discriminations, performance with the naturalistic setting was higher compared with that obtained with the Skinner box (*W* = 48, *P* = 0.002).

## Discussion

Guppies overall choose the reinforced numerosity significantly more often than chance, as indicated by the *t* test analysis. This confirms that Skinner-box-like devices can be used to investigate cognition in fish. However, the numerosity discrimination performance obtained by guppies appears rather low (range between 54 and 59% accuracy). It is not uncommon that fish significantly discriminate between two numerosities but exhibit low accuracy (e.g.,^23,28^), especially if compared with warm-blooded vertebrates, which often surpass 75% accuracy^29^. The latter gap might be associated to differences in the quantitative abilities of the species, but also to differences in other cognitive factors involved in the task, such as attention and decision-making abilities. However, it should be noted that in our study, the performance of guppies with the automatic conditioning chamber worsened compared to that observed with procedures based on naturalistic settings^9,24^. For example, Bisazza and colleagues^9^ found, with similar numerical ratios, an average accuracy of approximately 70% (range 60–83%). The modest performance in the present study is also underlined by a positive trend of accuracy over sessions and significant choice for the correct stimulus at the end of the training only for the ‘easier’ 0.6 numerical ratio. Finally, the poor numerical performance of our subjects was further indicated by the fact that above-chance performance was mainly due to the trials in which area and number were fully congruent. Therefore, guppies did not use pure numerical information to solve the task, but rather based their discrimination also on the continuous perceptual cues co-varying with number. This finding is in contrast with prior studies in which guppies proved able to solve incongruent numerosity discriminations^9,30^.

Interestingly, re-analysis of data from prior studies revealed that the low performance of guppies in the Skinner box is restricted to numerical discriminations (Fig. 4). For colour and shape discriminations, performance with the Skinner box was high and broadly overlapped that obtained with naturalistic training settings. For example, in a colour (red vs. yellow) discrimination with the Skinner box, guppies reached 80–90% accuracy after two days and in some sessions even achieved reached 100% correct choices, which is unusual for fish irrespective of the species, the type of discrimination and the procedure employed (e.g.,^23,27,31–33^).

Why do guppies tested with the Skinner-box paradigm show high performance in certain tasks and worsened performance in others? A simple explanation is that training in the Skinner box leads to high levels of performance in easy tasks, while for difficult tasks, performance gets poorer that in naturalistic settings. However, this explanation is based on the idea that numerical capacities are limited to few species with high levels of nervous system development. We now know that numerical capacities are widespread across animal species, including amphibians, fish, and invertebrates^5,6,34^. In addition, our analysis (Fig. 4) as well as prior studies^35^ indicate that fish can learn with similar efficiency a shape and a numerical discrimination.

It seems more likely that some characteristics of the Skinner box procedure (high number of trials, short inter-trial interval, constancy of reinforcement delay, etc.) could lead to a deterioration of performance in certain types of task, independently from their difficulty. The first factor to consider is cognitive load. Learning in our automated device requires the fish to initially acquire a series of additional skills, such as moving autonomously in a tank subdivided in specific (invisible) compartments, entering these compartments in the correct order and approaching the choice chambers to obtain food release. The cognitive load deriving from these simultaneous tasks might lower guppies’ discrimination performance, especially for the incongruent conditions^36^. In contrast, the paradigms in which fish achieved their greater numerical performance appear related to the natural behaviour of the species. Guppies and sticklebacks higher numerical acuity has been reported in the spontaneous choice of the larger social group^24,37^. Guppies also achieved their maximum accuracy in a paradigm whereby they selected between foraging patches with a different number of objects from which the food could be extracted^9^. In these natural-like settings, fish may not suffer the cognitive load of performing a set of learned behaviours, as it occurs in the Skinner box. Alternatively, specific cognitive modules may facilitate handling multiple simultaneous tasks in these natural situations. The cognitive load hypothesis deserves scrutiny in future studies. Indeed, the fact that guppies showed higher performance in the easy numerical discrimination task (ratio: 0.6) is partially in agreement with this hypothesis. An additional test would be training fish using naturalistic stimuli in the skinner box, such as images of conspecifics. Naturalistic stimuli might be processed more easily by fish, causing a reduction of the overall cognitive load. However, this test presents limitations. Although in some cases 2-D virtual images appears to trigger fish natural behaviour^38^, many concerns have been raised for their use because how animals perceive such virtual stimuli remains unclear (for a review^39^).

Another factor is related to numerosity discrimination in the Skinner box: the need to observe both and entirely the two stimuli. In a colour or shape discrimination, the same pair of stimuli is presented for the whole experiment. Therefore, the fish can solve the task by recognising one of the two stimuli or even a portion of a stimulus. The simple rule “approach the stimulus if it is the reinforced one and avoid it if is the non-reinforced one”^40,41^ should favour a habit and allow learning colour and shape discriminations in a Skinner-box condition. Conversely, in a numerosity discrimination task, each trial has a different pair of stimuli, because position, size, and density of the items vary systematically. Therefore, in each trial, the two new numerical stimuli must be fully seen, analysed and compared to take a decision. The relative quantity discrimination increases the attention necessary for taking a decision. In addition, it increases the time necessary to take the decision, which requires the subjects to inhibit the tendency to approach the choice area for much longer. In the Skinner box, where the trials rapidly succeed one another, fish might show difficulties in inhibiting the choice to take accurate decision. In line with this hypothesis, research on humans has indicated that inhibitory control is particularly relevant for discriminations in which non-numerical cues are incongruent with numerosity^42^, corresponding to the trials in which guppies achieved the lowest performance.

Overall, our results support the idea that part of the observed difference between the numerical abilities of fish and that of homeotherm vertebrates can be accounted by methodological factors. Nonetheless, this study highlighted that it is not always a matter of experimental setting, such as the number of training trials, but some of the factors involved may be cognitive. Both inhibitory control and attention vary considerably across species^43–45^. Species that possess greater capacities in these functions may be more attuned to achieve high performance in a Skinner box when the discrimination is difficult. Likewise, learning in the Skinner box may be favoured in species with more complex neural system because of the capacity to manage a greater cognitive load and complete more tasks simultaneously. For example, brain-imaging research in humans has suggested that the cortex, which fish do not possess, plays a critical role in cognitive multi-tasking^46,47^. Despite the cognitive functions responsible of numerical computation could be relatively similar across vertebrates, the performance assessment in a discrimination task is likely affected by differences in the whole cognitive system of the species.

## Supplementary information

Supplementary Table.

## Data Availability

Data have been submitted as supplementary material.

## References

[1] Boakes R (1984). From Darwin to Behaviourism: Psychology and the Minds of Animals.

[2] Dewsbury DA (1984). Comparative Psychology in the Twentieth Century.

[3] Washburn DA, Salamanca JA, Callery RC, Whitham W, Call J, Burghardt GM, Pepperberg IM, Snowdon CT, Zentall T (2017). Tools for measuring animal cognition: T mazes to touchscreens. APA Handbooks in Psychology. APA Handbook of Comparative Psychology: Basic Concepts, Methods, Neural Substrate, and Behaviour.

[4] Rose GJ (2018). The numerical abilities of anurans and their neural correlates: insights from neuroethological studies of acoustic communication. Philos. Trans. R. Soc. B. Biol. Sci..

[5] Agrillo C, Bisazza A (2018). Understanding the origin of number sense: a review of fish studies. Philos. Trans. R. Soc. B Biol Sci..

[6] Giurfa M (2019). An insect’s sense of number. Trends Cogn. Sci..

[7] Yang TI, Chiao CC (2016). Number sense and state-dependent valuation in cuttlefish. Proc. R. Soc. B. Biol. Sci..

[8] Lavigne DM (1982). Similarity in energy budgets of animal populations. J. Anim. Ecol..

[9] Bisazza A, Agrillo C, Lucon-Xiccato T (2014). Extensive training extends numerical abilities of guppies. Anim. Cogn..

[10] Miletto Petrazzini ME, Agrillo C, Izard V, Bisazza A (2015). Relative versus absolute numerical representation in fish: can guppies represent “fourness”?. Anim. Cogn..

[11] Miletto Petrazzini ME, Lucon-Xiccato T, Agrillo C, Bisazza A (2015). Use of ordinal information by fish. Sci. Rep..

[12] Beran MJ (2008). Monkeys (*Macaca mulatta* and *Cebus apella*) track, enumerate, and compare multiple sets of moving items. J. Exp. Psychol. Anim. Behav. Process..

[13] Emmerton J, Delius JD, Zeigler HP, Bischof HJ (1993). Beyond sensation: visual cognition in pigeons. Vision, Brain, and Behavior in Birds.

[14] Tomonaga M, Matsuzawa T (2002). Enumeration of briefly presented items by the chimpanzee (*Pan troglodytes*) and humans (*Homo sapiens*). Anim. Learn. Behav..

[15] DeLong CM, Barbato S, O’Leary T, Wilcox KT (2017). Small and large number discrimination in goldfish (*Carassius auratus*) with extensive training. Behav. Process..

[16] Lucon-Xiccato T, Manabe K, Bisazza A (2019). Guppies learn faster to discriminate between red and yellow than between two shapes. Ethology.

[17] Gatto E, Lucon-Xiccato T, Bisazza A, Manabe K, Dadda M (2020). The devil is in the detail: zebrafish learn to discriminate visual stimuli only if salient. Behav. Process..

[18] Agrillo C, Piffer L, Bisazza A (2010). Large number discrimination by mosquitofish. PLoS ONE.

[19] Zorzi M, Testolin A (2018). An emergentist perspective on the origin of number sense. Philos. Trans. R. Soc. B. Biol. Sci..

[20] Corral-López A, Garate-Olaizola M, Buechel SD, Kolm N, Kotrschal A (2017). On the role of body size, brain size, and eye size in visual acuity. Behav. Ecol. Sociobiol..

[21] Dadda M, Agrillo C, Bisazza A, Brown C (2015). Laterality enhances numerical skills in the guppy, *Poecilia reticulata*. Front. Behav. Neurosci..

[22] Piffer L, Agrillo C, Hyde DC (2012). Small and large number discrimination in guppies. Anim. Cogn..

[23] Agrillo C, Miletto Petrazzini ME, Tagliapietra C, Bisazza A (2012). Inter-specific differences in numerical abilities among teleost fish. Front. Psychol..

[24] Lucon-Xiccato T, Dadda M, Gatto E, Bisazza A (2017). Development and testing of a rapid method for measuring shoal size discrimination. Anim. Cogn..

[25] Testolin A, Dolfi S, Rochus M, Zorzi M (2020). Visual sense of number vs. sense of magnitude in humans and machines. Sci. Rep..

[26] Lucon-Xiccato T, Bisazza A (2014). Discrimination reversal learning reveals greater female behavioural flexibility in guppies. Biol. Lett..

[27] Lucon-Xiccato T, Bisazza A (2016). Male and female guppies differ in speed but not in accuracy in visual discrimination learning. Anim. Cogn..

[28] Bisazza A, Piffer L, Serena G, Agrillo C (2010). Ontogeny of numerical abilities in fish. PLoS ONE.

[29] Hanus D, Call J (2007). Discrete quantity judgments in the great apes (*Pan paniscus, Pan troglodytes, Gorilla gorilla, Pongo pygmaeus*): the effect of presenting whole sets versus item-by-item. J. Comp. Psychol..

[30] Agrillo C, Dadda M, Serena G, Bisazza A (2009). Use of number by fish. PLoS ONE.

[31] Colwill RM, Raymond MP, Ferreira L, Escudero H (2005). Visual discrimination learning in zebrafish (*Danio rerio*). Behav. Process..

[32] Siebeck UE, Litherland L, Wallis GM (2009). Shape learning and discrimination in reef fish. J. Exp. Biol..

[33] Wang MY, Brennan CH, Lachlan RF, Chittka L (2015). Speed–accuracy trade-offs and individually consistent decision making by individuals and dyads of zebrafish in a colour discrimination task. Anim. Behav..

[34] Lucon-Xiccato T, Gatto E, Bisazza A (2018). Quantity discrimination by treefrogs. Anim. Behav..

[35] Agrillo C, Piffer L, Bisazza A (2011). Number versus continuous quantity in numerosity judgments by fish. Cognition.

[36] Davis H, Pérusse R (1988). Numerical competence in animals: definitional issues, current evidence, and a new research agenda. Behav. Brain Sci..

[37] Mehlis M, Thünken T, Bakker TC, Frommen JG (2015). Quantification acuity in spontaneous shoaling decisions of three-spined sticklebacks. Anim. Cogn..

[38] Qin M, Wong A, Seguin D, Gerlai R (2014). Induction of social behavior in zebrafish: live versus computer animated fish as stimuli. Zebrafish.

[39] Chouinard-Thuly L, Gierszewski S, Rosenthal GG, Reader SM, Rieucau G, Woo KL, Gerlai R, Tedore C, Ingley SJ, Stowers JR, Frommen JG, Dolins FL, Witte K (2017). Technical and conceptual considerations for using animated stimuli in studies of animal behavior. Curr. Zool..

[40] Newport C, Wallis G, Temple SE, Siebeck UE (2013). Complex, context-dependent decision strategies of archerfish, *Toxotes chatareus*. Anim. Behav..

[41] Roembke TC, Wasserman EA, McMurray B (2016). Learning in rich networks involves both positive and negative associations. J. Exp. Psychol. Gen..

[42] Cappelletti M, Didino D, Stoianov I, Zorzi M (2014). Number skills are maintained in healthy ageing. Cogn. Psychol..

[43] Lucon-Xiccato T, Gatto E, Bisazza A (2017). Fish perform like mammals and birds in inhibitory motor control tasks. Sci. Rep..

[44] Kabadayi C, Taylor LA, von Bayern AM, Osvath M (2016). Ravens, New Caledonian crows and jackdaws parallel great apes in motor self-regulation despite smaller brains. R. Soc. Open Sci..

[45] MacLean E, Hare B, Nunn CL, Addessi E, Amici F, Anderson RC, Aureli F, Baker JM, Bania AE, Barnard AM, Boogert NJ, Brannon EM, Bray EE, Bray J, Brent LJN, Burkart JM, Call J, Cantlon JF, Cheke LG, Clayton NS, Delgado MM, DiVincenti LJ, Fujita K, Herrmann E, Hiramatsu C, Jacobs LF, Jordan KE, Laude JR, Leimgruber KL, Messer EJE, de Moura AC, Ostojić L, Picard A, Platt ML, Plotnik JM, Range F, Reader SM, Reddy RB, Sandel AA, Santos LR, Schumann K, Seed AM, Sewall KB, Shaw RC, Slocombe KE, Su Y, Takimoto A, Tan J, Tao R, van Schaik CP, Virányi Z, Visalberghi E, Wade JC, Watanabe A, Widness J, Young JK, Zentall TR, Zhao Y (2014). The evolution of self-control. Proc. Natl. Acad. Sci..

[46] Medeiros-Ward N, Watson JM, Strayer DL (2015). On supertaskers and the neural basis of efficient multitasking. Psychon. Bull. Rev..

[47] Verghese A, Garner KG, Mattingley JB, Dux PE (2016). Prefrontal cortex structure predicts training-induced improvements in multitasking performance. J. Neurosci..

