# Supplementary material for: Poor numerical performance of guppies tested in a Skinner box
Source: Sci Rep. 2020 Oct 7;10:16724. doi: 10.1038/s41598-020-73851-1 (PMC7542150; doi:10.1038/s41598-020-73851-1)
Supplement: Supplementary file 1 — Supplementary Table. [file 41598_2020_73851_MOESM1_ESM.pdf]

Poor numerical performance of guppies tested in a Skinner box

Elia Gatto, Alberto Testolin, Angelo Bisazza, Marco Zorzi, Tyrone Lucon-Xiccato

Table legend

Data collected in numerical training with the Skinner box. Each line represents a trial. The choice of the subject is reported as 1=correct choice, 0=incorrect choice. In addition, Subject ID, Type of correction, Numerical ratio and training Block are provided.

Subject_ID	Type_of_correction	Numerical_Ratio	Block	Choice
f01	Partially Congruent	r0.75	block01	0
f01	Partially Congruent	r0.75	block01	1
f01	Incogruent	r0.6	block01	0
f01	Fully Congruent	r0.75	block01	1
f01	Fully Congruent	r0.75	block01	0
f01	Partially Congruent	r0.75	block01	0
f01	Fully Congruent	r0.6	block01	1
f01	Incogruent	r0.75	block01	0
f01	Partially Congruent	r0.6	block01	1
f01	Partially Congruent	r0.6	block01	1
f01	Incogruent	r0.75	block01	1
f01	Partially Congruent	r0.6	block01	0
f01	Partially Congruent	r0.6	block01	1
f01	Partially Congruent	r0.6	block01	0
f01	Partially Congruent	r0.6	block01	1
f01	Incogruent	r0.75	block01	0
f01	Partially Congruent	r0.6	block01	0
f01	Partially Congruent	r0.75	block01	0
f01	Fully Congruent	r0.75	block01	1
f01	Fully Congruent	r0.75	block01	0
f01	Incogruent	r0.6	block01	0
f01	Incogruent	r0.75	block01	1
f01	Incogruent	r0.6	block01	0
f01	Fully Congruent	r0.6	block01	0
f01	Fully Congruent	r0.75	block01	0
f01	Partially Congruent	r0.6	block01	0
f01	Partially Congruent	r0.6	block01	0
f01	Partially Congruent	r0.6	block01	1
f01	Incogruent	r0.75	block01	0
f01	Incogruent	r0.6	block01	0
f01	Partially Congruent	r0.75	block01	0

f01	Incogruent	r0.6	block01	0
f01	Partially Congruent	r0.6	block01	1
f01	Partially Congruent	r0.6	block01	1
f01	Fully Congruent	r0.6	block01	0
f01	Partially Congruent	r0.6	block01	0
f01	Incogruent	r0.6	block01	1
f01	Incogruent	r0.6	block01	1
f01	Partially Congruent	r0.75	block01	1
f01	Partially Congruent	r0.75	block01	1
f01	Partially Congruent	r0.6	block01	1
f01	Incogruent	r0.6	block01	1
f01	Partially Congruent	r0.75	block01	0
f01	Fully Congruent	r0.75	block01	0
f01	Partially Congruent	r0.75	block01	0
f01	Partially Congruent	r0.6	block01	0
f01	Incogruent	r0.6	block01	0
f01	Incogruent	r0.6	block01	0
f01	Fully Congruent	r0.75	block01	1
f01	Partially Congruent	r0.75	block01	0
f01	Fully Congruent	r0.6	block02	1
f01	Fully Congruent	r0.6	block02	1
f01	Partially Congruent	r0.6	block02	1
f01	Incogruent	r0.6	block02	1
f01	Partially Congruent	r0.75	block02	0
f01	Fully Congruent	r0.6	block02	0
f01	Partially Congruent	r0.6	block02	0
f01	Partially Congruent	r0.75	block02	0
f01	Fully Congruent	r0.75	block02	1
f01	Incogruent	r0.6	block02	0
f01	Incogruent	r0.75	block02	1
f01	Incogruent	r0.6	block02	1
f01	Partially Congruent	r0.75	block02	0
f01	Fully Congruent	r0.6	block02	1
f01	Partially Congruent	r0.6	block02	1
f01	Fully Congruent	r0.75	block02	0
f01	Partially Congruent	r0.6	block02	0
f01	Incogruent	r0.75	block02	1
f01	Partially Congruent	r0.75	block02	0
f01	Fully Congruent	r0.6	block02	0
f01	Partially Congruent	r0.6	block02	1
f01	Incogruent	r0.75	block02	1
f01	Fully Congruent	r0.6	block02	1
f01	Partially Congruent	r0.6	block02	0
f01	Incogruent	r0.75	block02	1
f01	Partially Congruent	r0.6	block02	0

f01	Partially Congruent	r0.6	block02	1
f01	Incogruent	r0.6	block02	1
f01	Fully Congruent	r0.6	block02	1
f01	Partially Congruent	r0.75	block02	0
f01	Partially Congruent	r0.75	block02	1
f01	Partially Congruent	r0.6	block02	0
f01	Partially Congruent	r0.75	block02	0
f01	Incogruent	r0.75	block02	1
f01	Partially Congruent	r0.6	block02	1
f01	Partially Congruent	r0.6	block02	0
f01	Partially Congruent	r0.6	block02	0
f01	Incogruent	r0.6	block02	1
f01	Fully Congruent	r0.75	block02	1
f01	Partially Congruent	r0.6	block02	1
f01	Partially Congruent	r0.6	block02	1
f01	Fully Congruent	r0.75	block02	1
f01	Fully Congruent	r0.6	block02	0
f01	Fully Congruent	r0.75	block02	0
f01	Incogruent	r0.75	block02	0
f01	Partially Congruent	r0.6	block02	1
f01	Fully Congruent	r0.75	block02	1
f01	Incogruent	r0.75	block02	1
f01	Partially Congruent	r0.6	block02	0
f01	Partially Congruent	r0.75	block02	0
f01	Partially Congruent	r0.75	block03	1
f01	Partially Congruent	r0.75	block03	0
f01	Partially Congruent	r0.6	block03	0
f01	Fully Congruent	r0.6	block03	1
f01	Incogruent	r0.6	block03	0
f01	Incogruent	r0.75	block03	0
f01	Partially Congruent	r0.75	block03	1
f01	Fully Congruent	r0.75	block03	0
f01	Fully Congruent	r0.75	block03	0
f01	Fully Congruent	r0.75	block03	1
f01	Fully Congruent	r0.75	block03	0
f01	Partially Congruent	r0.6	block03	0
f01	Fully Congruent	r0.6	block03	0
f01	Partially Congruent	r0.6	block03	1
f01	Fully Congruent	r0.75	block03	0
f01	Fully Congruent	r0.6	block03	1
f01	Fully Congruent	r0.75	block03	0
f01	Fully Congruent	r0.75	block03	1
f01	Partially Congruent	r0.6	block03	1
f01	Partially Congruent	r0.6	block03	0
f01	Incogruent	r0.6	block03	1

f01	Incogruent	r0.75	block03	0
f01	Fully Congruent	r0.75	block03	1
f01	Partially Congruent	r0.75	block03	1
f01	Incogruent	r0.6	block03	1
f01	Incogruent	r0.6	block03	1
f01	Partially Congruent	r0.75	block03	1
f01	Partially Congruent	r0.75	block03	1
f01	Partially Congruent	r0.75	block03	0
f01	Partially Congruent	r0.6	block03	0
f01	Partially Congruent	r0.6	block03	1
f01	Partially Congruent	r0.75	block03	0
f01	Partially Congruent	r0.75	block03	1
f01	Fully Congruent	r0.75	block03	1
f01	Incogruent	r0.75	block03	0
f01	Partially Congruent	r0.6	block03	0
f01	Fully Congruent	r0.75	block03	1
f01	Fully Congruent	r0.75	block03	1
f01	Incogruent	r0.6	block03	0
f01	Partially Congruent	r0.6	block03	1
f01	Incogruent	r0.6	block03	1
f01	Partially Congruent	r0.6	block03	1
f01	Fully Congruent	r0.75	block03	1
f01	Incogruent	r0.6	block03	1
f01	Incogruent	r0.6	block03	1
f01	Incogruent	r0.75	block03	1
f01	Incogruent	r0.75	block03	1
f01	Incogruent	r0.75	block03	0
f01	Incogruent	r0.6	block03	0
f01	Fully Congruent	r0.75	block03	1
f01	Partially Congruent	r0.6	block04	1
f01	Partially Congruent	r0.75	block04	0
f01	Fully Congruent	r0.6	block04	1
f01	Fully Congruent	r0.75	block04	0
f01	Fully Congruent	r0.75	block04	1
f01	Partially Congruent	r0.6	block04	1
f01	Fully Congruent	r0.75	block04	1
f01	Incogruent	r0.6	block04	1
f01	Fully Congruent	r0.75	block04	1
f01	Fully Congruent	r0.75	block04	0
f01	Fully Congruent	r0.6	block04	1
f01	Fully Congruent	r0.6	block04	1
f01	Partially Congruent	r0.6	block04	1
f01	Incogruent	r0.75	block04	0
f01	Fully Congruent	r0.75	block04	0
f01	Fully Congruent	r0.75	block04	1

f01	Incogruent	r0.6	block04	1
f01	Partially Congruent	r0.75	block04	0
f01	Fully Congruent	r0.75	block04	1
f01	Partially Congruent	r0.6	block04	1
f01	Incogruent	r0.75	block04	0
f01	Fully Congruent	r0.75	block04	0
f01	Incogruent	r0.6	block04	1
f01	Partially Congruent	r0.6	block04	1
f01	Partially Congruent	r0.6	block04	0
f01	Incogruent	r0.75	block04	0
f01	Incogruent	r0.75	block04	1
f01	Incogruent	r0.6	block04	0
f01	Partially Congruent	r0.6	block04	0
f01	Fully Congruent	r0.75	block04	0
f01	Fully Congruent	r0.6	block04	1
f01	Fully Congruent	r0.75	block04	1
f01	Partially Congruent	r0.6	block04	0
f01	Incogruent	r0.6	block04	0
f01	Fully Congruent	r0.75	block04	1
f01	Fully Congruent	r0.6	block04	1
f01	Incogruent	r0.6	block04	1
f01	Fully Congruent	r0.75	block04	1
f01	Partially Congruent	r0.6	block04	0
f01	Fully Congruent	r0.6	block04	0
f01	Incogruent	r0.6	block04	1
f01	Incogruent	r0.6	block04	1
f01	Fully Congruent	r0.75	block04	1
f01	Incogruent	r0.75	block04	0
f01	Partially Congruent	r0.75	block04	1
f01	Incogruent	r0.6	block04	1
f01	Fully Congruent	r0.75	block04	1
f01	Partially Congruent	r0.6	block04	1
f01	Fully Congruent	r0.75	block04	1
f01	Fully Congruent	r0.75	block04	1
f01	Partially Congruent	r0.6	block05	0
f01	Incogruent	r0.6	block05	1
f01	Partially Congruent	r0.75	block05	0
f01	Fully Congruent	r0.75	block05	1
f01	Incogruent	r0.6	block05	0
f01	Fully Congruent	r0.75	block05	1
f01	Incogruent	r0.75	block05	1
f01	Incogruent	r0.6	block05	0
f01	Fully Congruent	r0.6	block05	0
f01	Fully Congruent	r0.6	block05	1
f01	Fully Congruent	r0.75	block05	1

f01	Partially Congruent	r0.6	block05	0
f01	Partially Congruent	r0.6	block05	0
f01	Incogruent	r0.6	block05	1
f01	Fully Congruent	r0.6	block05	0
f01	Partially Congruent	r0.6	block05	1
f01	Fully Congruent	r0.75	block05	0
f01	Partially Congruent	r0.75	block05	0
f01	Incogruent	r0.6	block05	1
f01	Partially Congruent	r0.6	block05	0
f01	Fully Congruent	r0.75	block05	1
f01	Incogruent	r0.75	block05	1
f01	Incogruent	r0.6	block05	0
f01	Partially Congruent	r0.6	block05	0
f01	Partially Congruent	r0.6	block05	1
f01	Incogruent	r0.75	block05	0
f01	Fully Congruent	r0.75	block05	1
f01	Fully Congruent	r0.75	block05	1
f01	Incogruent	r0.75	block05	1
f01	Fully Congruent	r0.6	block05	1
f01	Partially Congruent	r0.6	block05	1
f01	Partially Congruent	r0.6	block05	0
f01	Partially Congruent	r0.75	block05	0
f01	Partially Congruent	r0.6	block05	1
f01	Incogruent	r0.6	block05	0
f01	Partially Congruent	r0.6	block05	1
f01	Fully Congruent	r0.75	block05	1
f01	Partially Congruent	r0.75	block05	0
f01	Incogruent	r0.6	block05	0
f01	Incogruent	r0.6	block05	0
f01	Partially Congruent	r0.75	block05	0
f01	Incogruent	r0.75	block05	1
f01	Incogruent	r0.75	block05	1
f01	Partially Congruent	r0.75	block05	1
f01	Incogruent	r0.6	block05	1
f01	Partially Congruent	r0.6	block05	1
f01	Fully Congruent	r0.75	block05	1
f01	Partially Congruent	r0.6	block05	0
f01	Partially Congruent	r0.75	block05	0
f01	Partially Congruent	r0.6	block05	1
f01	Incogruent	r0.6	block06	1
f01	Partially Congruent	r0.75	block06	1
f01	Fully Congruent	r0.75	block06	1
f01	Fully Congruent	r0.75	block06	1
f01	Fully Congruent	r0.6	block06	1
f01	Fully Congruent	r0.6	block06	0

f01	Incogruent	r0.6	block06	1
f01	Incogruent	r0.75	block06	0
f01	Fully Congruent	r0.75	block06	0
f01	Incogruent	r0.6	block06	0
f01	Fully Congruent	r0.75	block06	0
f01	Partially Congruent	r0.6	block06	0
f01	Partially Congruent	r0.75	block06	1
f01	Fully Congruent	r0.75	block06	1
f01	Fully Congruent	r0.6	block06	1
f01	Incogruent	r0.6	block06	0
f01	Incogruent	r0.75	block06	0
f01	Partially Congruent	r0.75	block06	1
f01	Fully Congruent	r0.6	block06	0
f01	Partially Congruent	r0.6	block06	0
f01	Incogruent	r0.75	block06	0
f01	Partially Congruent	r0.75	block06	1
f01	Incogruent	r0.6	block06	0
f01	Incogruent	r0.75	block06	1
f01	Fully Congruent	r0.6	block06	1
f01	Fully Congruent	r0.75	block06	1
f01	Incogruent	r0.75	block06	1
f01	Incogruent	r0.6	block06	0
f01	Fully Congruent	r0.75	block06	1
f01	Partially Congruent	r0.6	block06	0
f01	Partially Congruent	r0.6	block06	0
f01	Partially Congruent	r0.6	block06	1
f01	Partially Congruent	r0.75	block06	1
f01	Fully Congruent	r0.6	block06	1
f01	Fully Congruent	r0.75	block06	1
f01	Incogruent	r0.6	block06	1
f01	Fully Congruent	r0.6	block06	1
f01	Partially Congruent	r0.75	block06	1
f01	Incogruent	r0.75	block06	0
f01	Partially Congruent	r0.75	block06	0
f01	Fully Congruent	r0.75	block06	1
f01	Incogruent	r0.6	block06	1
f01	Fully Congruent	r0.6	block06	1
f01	Fully Congruent	r0.75	block06	1
f01	Partially Congruent	r0.6	block06	1
f01	Partially Congruent	r0.6	block06	0
f01	Fully Congruent	r0.6	block06	1
f01	Partially Congruent	r0.6	block06	0
f01	Incogruent	r0.6	block06	0
f01	Fully Congruent	r0.75	block06	1
f01	Fully Congruent	r0.75	block07	1

f01	Incogruent	r0.75	block07	0
f01	Partially Congruent	r0.75	block07	0
f01	Fully Congruent	r0.75	block07	1
f01	Fully Congruent	r0.75	block07	1
f01	Fully Congruent	r0.75	block07	1
f01	Incogruent	r0.75	block07	1
f01	Fully Congruent	r0.75	block07	0
f01	Partially Congruent	r0.6	block07	0
f01	Fully Congruent	r0.75	block07	0
f01	Fully Congruent	r0.75	block07	1
f01	Incogruent	r0.6	block07	0
f01	Partially Congruent	r0.75	block07	1
f01	Fully Congruent	r0.75	block07	0
f01	Incogruent	r0.6	block07	1
f01	Partially Congruent	r0.6	block07	1
f01	Partially Congruent	r0.75	block07	1
f01	Incogruent	r0.75	block07	1
f01	Partially Congruent	r0.75	block07	1
f01	Fully Congruent	r0.6	block07	0
f01	Partially Congruent	r0.75	block07	1
f01	Partially Congruent	r0.75	block07	1
f01	Partially Congruent	r0.6	block07	0
f01	Fully Congruent	r0.6	block07	1
f01	Incogruent	r0.6	block07	1
f01	Incogruent	r0.6	block07	1
f01	Fully Congruent	r0.75	block07	1
f01	Partially Congruent	r0.75	block07	1
f01	Incogruent	r0.6	block07	1
f01	Fully Congruent	r0.75	block07	1
f01	Partially Congruent	r0.6	block07	0
f01	Partially Congruent	r0.6	block07	1
f01	Fully Congruent	r0.75	block07	1
f01	Partially Congruent	r0.6	block07	1
f01	Incogruent	r0.75	block07	0
f01	Partially Congruent	r0.75	block07	0
f01	Fully Congruent	r0.75	block07	0
f01	Fully Congruent	r0.75	block07	1
f01	Incogruent	r0.6	block07	1
f01	Fully Congruent	r0.75	block07	0
f01	Incogruent	r0.75	block07	0
f01	Partially Congruent	r0.75	block07	0
f01	Incogruent	r0.75	block07	1
f01	Incogruent	r0.6	block07	1
f01	Partially Congruent	r0.6	block07	0
f01	Fully Congruent	r0.75	block07	0

f01	Incogruent	r0.75	block07	1
f01	Fully Congruent	r0.75	block07	1
f01	Incogruent	r0.6	block07	1
f01	Partially Congruent	r0.6	block07	0
f01	Partially Congruent	r0.6	block08	1
f01	Partially Congruent	r0.6	block08	0
f01	Incogruent	r0.6	block08	0
f01	Fully Congruent	r0.75	block08	1
f01	Partially Congruent	r0.6	block08	0
f01	Fully Congruent	r0.75	block08	0
f01	Partially Congruent	r0.6	block08	1
f01	Fully Congruent	r0.75	block08	1
f01	Partially Congruent	r0.75	block08	0
f01	Fully Congruent	r0.6	block08	1
f01	Incogruent	r0.75	block08	0
f01	Incogruent	r0.6	block08	1
f01	Partially Congruent	r0.6	block08	1
f01	Partially Congruent	r0.75	block08	0
f01	Incogruent	r0.6	block08	1
f01	Fully Congruent	r0.6	block08	1
f01	Incogruent	r0.6	block08	0
f01	Partially Congruent	r0.75	block08	0
f01	Incogruent	r0.75	block08	1
f01	Partially Congruent	r0.6	block08	1
f01	Fully Congruent	r0.75	block08	1
f01	Partially Congruent	r0.75	block08	0
f01	Incogruent	r0.75	block08	1
f01	Incogruent	r0.75	block08	1
f01	Partially Congruent	r0.75	block08	1
f01	Fully Congruent	r0.75	block08	1
f01	Fully Congruent	r0.75	block08	1
f01	Incogruent	r0.75	block08	1
f01	Fully Congruent	r0.6	block08	1
f01	Fully Congruent	r0.75	block08	0
f01	Partially Congruent	r0.75	block08	1
f01	Incogruent	r0.6	block08	1
f01	Fully Congruent	r0.6	block08	1
f01	Incogruent	r0.6	block08	1
f01	Partially Congruent	r0.75	block08	0
f01	Partially Congruent	r0.6	block08	0
f01	Incogruent	r0.6	block08	1
f01	Fully Congruent	r0.75	block08	1
f01	Fully Congruent	r0.75	block08	1
f01	Partially Congruent	r0.6	block08	1
f01	Fully Congruent	r0.75	block08	1

f01	Incogruent	r0.6	block08	0
f01	Incogruent	r0.75	block08	0
f01	Fully Congruent	r0.75	block08	1
f01	Fully Congruent	r0.75	block08	0
f01	Fully Congruent	r0.75	block08	1
f01	Incogruent	r0.75	block08	0
f01	Incogruent	r0.6	block08	1
f01	Fully Congruent	r0.6	block08	1
f01	Incogruent	r0.6	block08	0
f01	Fully Congruent	r0.6	block09	1
f01	Partially Congruent	r0.6	block09	0
f01	Fully Congruent	r0.75	block09	1
f01	Incogruent	r0.75	block09	0
f01	Partially Congruent	r0.6	block09	0
f01	Fully Congruent	r0.75	block09	1
f01	Partially Congruent	r0.75	block09	0
f01	Fully Congruent	r0.6	block09	0
f01	Fully Congruent	r0.75	block09	1
f01	Incogruent	r0.75	block09	0
f01	Incogruent	r0.6	block09	1
f01	Partially Congruent	r0.75	block09	1
f01	Fully Congruent	r0.75	block09	1
f01	Fully Congruent	r0.75	block09	1
f01	Partially Congruent	r0.75	block09	1
f01	Partially Congruent	r0.6	block09	0
f01	Partially Congruent	r0.75	block09	1
f01	Partially Congruent	r0.6	block09	1
f01	Fully Congruent	r0.75	block09	0
f01	Incogruent	r0.75	block09	0
f01	Partially Congruent	r0.75	block09	0
f01	Fully Congruent	r0.75	block09	1
f01	Fully Congruent	r0.75	block09	0
f01	Fully Congruent	r0.75	block09	1
f01	Incogruent	r0.75	block09	1
f01	Incogruent	r0.6	block09	1
f01	Fully Congruent	r0.75	block09	1
f01	Incogruent	r0.75	block09	1
f01	Incogruent	r0.75	block09	1
f01	Incogruent	r0.6	block09	1
f01	Incogruent	r0.6	block09	0
f01	Incogruent	r0.6	block09	1
f01	Incogruent	r0.6	block09	1
f01	Fully Congruent	r0.75	block09	1
f01	Partially Congruent	r0.75	block09	0
f01	Partially Congruent	r0.6	block09	1

f01	Incogruent	r0.75	block09	1
f01	Fully Congruent	r0.75	block09	1
f01	Fully Congruent	r0.75	block09	0
f01	Fully Congruent	r0.6	block09	1
f01	Partially Congruent	r0.6	block09	1
f01	Partially Congruent	r0.75	block09	1
f01	Partially Congruent	r0.75	block09	0
f01	Fully Congruent	r0.75	block09	1
f01	Partially Congruent	r0.6	block09	0
f01	Partially Congruent	r0.6	block09	1
f01	Partially Congruent	r0.6	block09	0
f01	Incogruent	r0.6	block09	1
f01	Fully Congruent	r0.75	block09	1
f01	Incogruent	r0.6	block09	1
f01	Incogruent	r0.6	block10	1
f01	Fully Congruent	r0.75	block10	1
f01	Partially Congruent	r0.75	block10	0
f01	Partially Congruent	r0.6	block10	0
f01	Incogruent	r0.6	block10	1
f01	Partially Congruent	r0.75	block10	0
f01	Partially Congruent	r0.6	block10	0
f01	Fully Congruent	r0.6	block10	0
f01	Fully Congruent	r0.6	block10	1
f01	Incogruent	r0.75	block10	1
f01	Fully Congruent	r0.6	block10	1
f01	Fully Congruent	r0.75	block10	1
f01	Fully Congruent	r0.75	block10	0
f01	Partially Congruent	r0.75	block10	0
f01	Partially Congruent	r0.75	block10	1
f01	Partially Congruent	r0.6	block10	0
f01	Incogruent	r0.75	block10	0
f01	Incogruent	r0.75	block10	0
f01	Partially Congruent	r0.75	block10	1
f01	Incogruent	r0.6	block10	1
f01	Incogruent	r0.6	block10	0
f01	Partially Congruent	r0.6	block10	1
f01	Partially Congruent	r0.6	block10	0
f01	Partially Congruent	r0.6	block10	1
f01	Incogruent	r0.6	block10	1
f01	Incogruent	r0.6	block10	1
f01	Incogruent	r0.75	block10	1
f01	Incogruent	r0.6	block10	1
f01	Partially Congruent	r0.6	block10	0
f01	Fully Congruent	r0.75	block10	1
f01	Partially Congruent	r0.75	block10	1

f01	Fully Congruent	r0.6	block10	1
f01	Incogruent	r0.6	block10	0
f01	Partially Congruent	r0.6	block10	1
f01	Incogruent	r0.6	block10	1
f01	Incogruent	r0.75	block10	0
f01	Incogruent	r0.75	block10	1
f01	Incogruent	r0.75	block10	1
f01	Incogruent	r0.75	block10	0
f01	Partially Congruent	r0.75	block10	0
f01	Fully Congruent	r0.6	block10	1
f01	Incogruent	r0.75	block10	0
f01	Fully Congruent	r0.75	block10	1
f01	Fully Congruent	r0.75	block10	1
f01	Fully Congruent	r0.6	block10	1
f01	Incogruent	r0.75	block10	1
f01	Fully Congruent	r0.6	block10	1
f01	Partially Congruent	r0.6	block10	1
f01	Partially Congruent	r0.6	block10	1
f01	Fully Congruent	r0.75	block10	1
f04	Fully Congruent	r0.75	block01	1
f04	Incogruent	r0.75	block01	1
f04	Partially Congruent	r0.75	block01	1
f04	Partially Congruent	r0.75	block01	0
f04	Partially Congruent	r0.75	block01	1
f04	Fully Congruent	r0.75	block01	1
f04	Partially Congruent	r0.6	block01	0
f04	Partially Congruent	r0.6	block01	1
f04	Fully Congruent	r0.6	block01	1
f04	Incogruent	r0.75	block01	0
f04	Fully Congruent	r0.75	block01	1
f04	Incogruent	r0.6	block01	1
f04	Fully Congruent	r0.75	block01	1
f04	Fully Congruent	r0.75	block01	1
f04	Partially Congruent	r0.75	block01	0
f04	Fully Congruent	r0.6	block01	0
f04	Fully Congruent	r0.75	block01	0
f04	Partially Congruent	r0.75	block01	1
f04	Fully Congruent	r0.6	block01	0
f04	Fully Congruent	r0.75	block01	1
f04	Partially Congruent	r0.75	block01	1
f04	Fully Congruent	r0.6	block01	1
f04	Fully Congruent	r0.75	block01	1
f04	Incogruent	r0.75	block01	0
f04	Partially Congruent	r0.6	block01	0
f04	Partially Congruent	r0.6	block01	0

f04	Fully Congruent	r0.75	block01	1
f04	Partially Congruent	r0.75	block01	0
f04	Fully Congruent	r0.6	block01	1
f04	Incogruent	r0.6	block01	0
f04	Incogruent	r0.75	block01	0
f04	Partially Congruent	r0.6	block01	0
f04	Partially Congruent	r0.6	block01	0
f04	Incogruent	r0.6	block01	0
f04	Partially Congruent	r0.6	block01	1
f04	Partially Congruent	r0.6	block01	1
f04	Incogruent	r0.75	block01	1
f04	Incogruent	r0.75	block01	0
f04	Fully Congruent	r0.75	block01	0
f04	Fully Congruent	r0.75	block01	0
f04	Partially Congruent	r0.6	block01	1
f04	Fully Congruent	r0.75	block01	0
f04	Incogruent	r0.6	block01	1
f04	Fully Congruent	r0.75	block01	1
f04	Partially Congruent	r0.6	block01	0
f04	Partially Congruent	r0.6	block01	0
f04	Fully Congruent	r0.6	block01	0
f04	Partially Congruent	r0.6	block01	0
f04	Fully Congruent	r0.75	block01	0
f04	Incogruent	r0.6	block01	0
f04	Partially Congruent	r0.6	block02	0
f04	Fully Congruent	r0.75	block02	0
f04	Fully Congruent	r0.75	block02	0
f04	Fully Congruent	r0.75	block02	1
f04	Fully Congruent	r0.75	block02	1
f04	Partially Congruent	r0.6	block02	0
f04	Partially Congruent	r0.75	block02	0
f04	Incogruent	r0.75	block02	1
f04	Fully Congruent	r0.6	block02	0
f04	Incogruent	r0.6	block02	1
f04	Incogruent	r0.6	block02	0
f04	Incogruent	r0.6	block02	1
f04	Fully Congruent	r0.75	block02	1
f04	Fully Congruent	r0.75	block02	1
f04	Partially Congruent	r0.75	block02	0
f04	Incogruent	r0.75	block02	1
f04	Fully Congruent	r0.75	block02	1
f04	Partially Congruent	r0.75	block02	1
f04	Partially Congruent	r0.6	block02	0
f04	Fully Congruent	r0.75	block02	0
f04	Incogruent	r0.75	block02	1

f04	Partially Congruent	r0.6	block02	1
f04	Partially Congruent	r0.6	block02	0
f04	Fully Congruent	r0.6	block02	1
f04	Fully Congruent	r0.75	block02	0
f04	Incogruent	r0.75	block02	0
f04	Fully Congruent	r0.6	block02	0
f04	Fully Congruent	r0.75	block02	1
f04	Partially Congruent	r0.75	block02	1
f04	Partially Congruent	r0.6	block02	1
f04	Partially Congruent	r0.6	block02	0
f04	Partially Congruent	r0.6	block02	1
f04	Fully Congruent	r0.75	block02	1
f04	Fully Congruent	r0.75	block02	1
f04	Partially Congruent	r0.75	block02	0
f04	Fully Congruent	r0.75	block02	0
f04	Incogruent	r0.6	block02	0
f04	Fully Congruent	r0.75	block02	1
f04	Incogruent	r0.6	block02	1
f04	Incogruent	r0.75	block02	0
f04	Fully Congruent	r0.75	block02	0
f04	Incogruent	r0.75	block02	1
f04	Incogruent	r0.75	block02	1
f04	Fully Congruent	r0.6	block02	0
f04	Incogruent	r0.6	block02	0
f04	Incogruent	r0.6	block02	1
f04	Fully Congruent	r0.75	block02	1
f04	Partially Congruent	r0.6	block02	1
f04	Fully Congruent	r0.6	block02	1
f04	Incogruent	r0.6	block02	0
f04	Fully Congruent	r0.75	block03	1
f04	Fully Congruent	r0.6	block03	0
f04	Partially Congruent	r0.75	block03	0
f04	Incogruent	r0.6	block03	1
f04	Fully Congruent	r0.75	block03	1
f04	Fully Congruent	r0.75	block03	0
f04	Fully Congruent	r0.75	block03	1
f04	Fully Congruent	r0.6	block03	1
f04	Partially Congruent	r0.6	block03	1
f04	Fully Congruent	r0.75	block03	1
f04	Fully Congruent	r0.6	block03	1
f04	Fully Congruent	r0.75	block03	0
f04	Fully Congruent	r0.6	block03	1
f04	Incogruent	r0.6	block03	0
f04	Partially Congruent	r0.75	block03	0
f04	Fully Congruent	r0.75	block03	1

f04	Fully Congruent	r0.6	block03	0
f04	Partially Congruent	r0.6	block03	1
f04	Fully Congruent	r0.75	block03	0
f04	Fully Congruent	r0.6	block03	1
f04	Fully Congruent	r0.75	block03	1
f04	Fully Congruent	r0.6	block03	1
f04	Partially Congruent	r0.6	block03	1
f04	Incogruent	r0.6	block03	0
f04	Fully Congruent	r0.75	block03	0
f04	Fully Congruent	r0.6	block03	1
f04	Fully Congruent	r0.75	block03	1
f04	Partially Congruent	r0.75	block03	1
f04	Fully Congruent	r0.75	block03	1
f04	Partially Congruent	r0.75	block03	1
f04	Incogruent	r0.75	block03	1
f04	Incogruent	r0.75	block03	0
f04	Partially Congruent	r0.75	block03	1
f04	Fully Congruent	r0.75	block03	1
f04	Incogruent	r0.6	block03	1
f04	Incogruent	r0.75	block03	1
f04	Partially Congruent	r0.6	block03	0
f04	Incogruent	r0.75	block03	0
f04	Partially Congruent	r0.6	block03	0
f04	Fully Congruent	r0.6	block03	1
f04	Fully Congruent	r0.75	block03	1
f04	Partially Congruent	r0.75	block03	1
f04	Fully Congruent	r0.75	block03	1
f04	Fully Congruent	r0.6	block03	0
f04	Incogruent	r0.6	block03	0
f04	Partially Congruent	r0.75	block03	0
f04	Fully Congruent	r0.75	block03	0
f04	Partially Congruent	r0.6	block03	1
f04	Partially Congruent	r0.6	block03	0
f04	Fully Congruent	r0.75	block03	1
f04	Partially Congruent	r0.6	block04	0
f04	Partially Congruent	r0.6	block04	0
f04	Fully Congruent	r0.6	block04	0
f04	Fully Congruent	r0.75	block04	1
f04	Partially Congruent	r0.6	block04	0
f04	Fully Congruent	r0.75	block04	0
f04	Incogruent	r0.6	block04	1
f04	Incogruent	r0.75	block04	1
f04	Fully Congruent	r0.6	block04	1
f04	Incogruent	r0.6	block04	1
f04	Fully Congruent	r0.6	block04	1

f04	Partially Congruent	r0.6	block04	0
f04	Fully Congruent	r0.75	block04	0
f04	Fully Congruent	r0.75	block04	1
f04	Partially Congruent	r0.6	block04	0
f04	Incogruent	r0.6	block04	0
f04	Fully Congruent	r0.75	block04	1
f04	Partially Congruent	r0.75	block04	1
f04	Fully Congruent	r0.75	block04	0
f04	Incogruent	r0.6	block04	1
f04	Incogruent	r0.75	block04	1
f04	Incogruent	r0.75	block04	1
f04	Incogruent	r0.6	block04	0
f04	Incogruent	r0.6	block04	1
f04	Partially Congruent	r0.75	block04	1
f04	Fully Congruent	r0.75	block04	1
f04	Partially Congruent	r0.6	block04	0
f04	Partially Congruent	r0.6	block04	1
f04	Incogruent	r0.6	block04	0
f04	Fully Congruent	r0.75	block04	1
f04	Incogruent	r0.75	block04	0
f04	Fully Congruent	r0.6	block04	0
f04	Fully Congruent	r0.6	block04	1
f04	Partially Congruent	r0.75	block04	0
f04	Fully Congruent	r0.75	block04	1
f04	Incogruent	r0.6	block04	0
f04	Partially Congruent	r0.6	block04	1
f04	Fully Congruent	r0.75	block04	1
f04	Fully Congruent	r0.6	block04	1
f04	Partially Congruent	r0.75	block04	1
f04	Fully Congruent	r0.6	block04	1
f04	Partially Congruent	r0.6	block04	0
f04	Incogruent	r0.75	block04	0
f04	Fully Congruent	r0.75	block04	1
f04	Partially Congruent	r0.6	block04	0
f04	Incogruent	r0.6	block04	1
f04	Incogruent	r0.75	block04	0
f04	Partially Congruent	r0.75	block04	1
f04	Incogruent	r0.6	block04	0
f04	Incogruent	r0.6	block04	1
f04	Incogruent	r0.75	block05	0
f04	Incogruent	r0.75	block05	0
f04	Fully Congruent	r0.75	block05	1
f04	Partially Congruent	r0.75	block05	0
f04	Fully Congruent	r0.6	block05	1
f04	Incogruent	r0.75	block05	0

f04	Partially Congruent	r0.6	block05	1
f04	Incogruent	r0.6	block05	0
f04	Partially Congruent	r0.6	block05	0
f04	Partially Congruent	r0.6	block05	1
f04	Fully Congruent	r0.6	block05	1
f04	Incogruent	r0.75	block05	1
f04	Partially Congruent	r0.6	block05	1
f04	Fully Congruent	r0.6	block05	1
f04	Fully Congruent	r0.75	block05	1
f04	Incogruent	r0.6	block05	0
f04	Fully Congruent	r0.75	block05	1
f04	Partially Congruent	r0.6	block05	1
f04	Fully Congruent	r0.75	block05	0
f04	Partially Congruent	r0.75	block05	0
f04	Incogruent	r0.75	block05	1
f04	Partially Congruent	r0.6	block05	1
f04	Fully Congruent	r0.6	block05	0
f04	Partially Congruent	r0.6	block05	1
f04	Partially Congruent	r0.6	block05	0
f04	Incogruent	r0.75	block05	0
f04	Fully Congruent	r0.6	block05	1
f04	Partially Congruent	r0.6	block05	0
f04	Partially Congruent	r0.75	block05	1
f04	Partially Congruent	r0.6	block05	0
f04	Incogruent	r0.75	block05	0
f04	Incogruent	r0.6	block05	0
f04	Partially Congruent	r0.6	block05	0
f04	Partially Congruent	r0.6	block05	1
f04	Fully Congruent	r0.75	block05	1
f04	Partially Congruent	r0.6	block05	0
f04	Incogruent	r0.75	block05	0
f04	Fully Congruent	r0.75	block05	1
f04	Partially Congruent	r0.75	block05	0
f04	Partially Congruent	r0.75	block05	1
f04	Fully Congruent	r0.75	block05	1
f04	Partially Congruent	r0.6	block05	0
f04	Incogruent	r0.6	block05	1
f04	Incogruent	r0.6	block05	0
f04	Partially Congruent	r0.6	block05	0
f04	Partially Congruent	r0.6	block05	0
f04	Fully Congruent	r0.75	block05	1
f04	Fully Congruent	r0.75	block05	1
f04	Fully Congruent	r0.75	block05	1
f04	Fully Congruent	r0.6	block05	1
f04	Fully Congruent	r0.6	block06	1

f04	Incogruent	r0.75	block06	0
f04	Fully Congruent	r0.75	block06	0
f04	Fully Congruent	r0.75	block06	1
f04	Fully Congruent	r0.75	block06	0
f04	Partially Congruent	r0.75	block06	0
f04	Partially Congruent	r0.6	block06	1
f04	Partially Congruent	r0.6	block06	1
f04	Fully Congruent	r0.75	block06	1
f04	Fully Congruent	r0.75	block06	1
f04	Fully Congruent	r0.6	block06	1
f04	Partially Congruent	r0.6	block06	1
f04	Fully Congruent	r0.75	block06	1
f04	Incogruent	r0.6	block06	1
f04	Fully Congruent	r0.75	block06	1
f04	Fully Congruent	r0.75	block06	1
f04	Incogruent	r0.75	block06	1
f04	Incogruent	r0.6	block06	0
f04	Incogruent	r0.75	block06	0
f04	Fully Congruent	r0.75	block06	1
f04	Fully Congruent	r0.75	block06	1
f04	Partially Congruent	r0.75	block06	1
f04	Partially Congruent	r0.6	block06	0
f04	Fully Congruent	r0.75	block06	0
f04	Incogruent	r0.6	block06	1
f04	Incogruent	r0.75	block06	1
f04	Partially Congruent	r0.75	block06	1
f04	Partially Congruent	r0.6	block06	0
f04	Fully Congruent	r0.75	block06	0
f04	Incogruent	r0.6	block06	1
f04	Incogruent	r0.75	block06	0
f04	Fully Congruent	r0.75	block06	1
f04	Partially Congruent	r0.6	block06	0
f04	Partially Congruent	r0.6	block06	1
f04	Fully Congruent	r0.75	block06	1
f04	Fully Congruent	r0.75	block06	1
f04	Incogruent	r0.6	block06	0
f04	Fully Congruent	r0.75	block06	0
f04	Incogruent	r0.6	block06	1
f04	Partially Congruent	r0.6	block06	0
f04	Partially Congruent	r0.6	block06	0
f04	Partially Congruent	r0.75	block06	0
f04	Partially Congruent	r0.75	block06	1
f04	Partially Congruent	r0.75	block06	1
f04	Incogruent	r0.75	block06	1
f04	Partially Congruent	r0.6	block06	0

f04	Partially Congruent	r0.6	block06	0
f04	Incogruent	r0.6	block06	1
f04	Incogruent	r0.6	block06	1
f04	Incogruent	r0.6	block06	0
f04	Partially Congruent	r0.6	block07	0
f04	Incogruent	r0.6	block07	0
f04	Incogruent	r0.75	block07	0
f04	Incogruent	r0.6	block07	0
f04	Fully Congruent	r0.6	block07	1
f04	Fully Congruent	r0.6	block07	1
f04	Partially Congruent	r0.75	block07	0
f04	Partially Congruent	r0.6	block07	0
f04	Fully Congruent	r0.6	block07	0
f04	Partially Congruent	r0.6	block07	1
f04	Partially Congruent	r0.75	block07	0
f04	Fully Congruent	r0.75	block07	0
f04	Fully Congruent	r0.6	block07	1
f04	Fully Congruent	r0.75	block07	0
f04	Partially Congruent	r0.75	block07	1
f04	Incogruent	r0.75	block07	0
f04	Incogruent	r0.6	block07	0
f04	Incogruent	r0.6	block07	1
f04	Fully Congruent	r0.75	block07	1
f04	Incogruent	r0.75	block07	0
f04	Partially Congruent	r0.75	block07	1
f04	Incogruent	r0.6	block07	0
f04	Incogruent	r0.75	block07	0
f04	Partially Congruent	r0.6	block07	1
f04	Incogruent	r0.6	block07	1
f04	Incogruent	r0.75	block07	0
f04	Incogruent	r0.6	block07	1
f04	Partially Congruent	r0.75	block07	1
f04	Incogruent	r0.6	block07	0
f04	Fully Congruent	r0.75	block07	1
f04	Incogruent	r0.6	block07	1
f04	Fully Congruent	r0.75	block07	0
f04	Incogruent	r0.75	block07	0
f04	Fully Congruent	r0.6	block07	1
f04	Partially Congruent	r0.6	block07	0
f04	Incogruent	r0.6	block07	1
f04	Partially Congruent	r0.6	block07	1
f04	Incogruent	r0.6	block07	1
f04	Partially Congruent	r0.6	block07	1
f04	Incogruent	r0.75	block07	1
f04	Incogruent	r0.75	block07	1

f04	Partially Congruent	r0.6	block07	1
f04	Incogruent	r0.6	block07	0
f04	Incogruent	r0.6	block07	0
f04	Fully Congruent	r0.75	block07	1
f04	Fully Congruent	r0.75	block07	0
f04	Partially Congruent	r0.6	block07	0
f04	Partially Congruent	r0.6	block07	0
f04	Fully Congruent	r0.6	block07	1
f04	Fully Congruent	r0.6	block07	1
f04	Fully Congruent	r0.75	block08	0
f04	Fully Congruent	r0.6	block08	1
f04	Fully Congruent	r0.6	block08	1
f04	Fully Congruent	r0.75	block08	1
f04	Fully Congruent	r0.75	block08	1
f04	Incogruent	r0.75	block08	0
f04	Incogruent	r0.75	block08	0
f04	Partially Congruent	r0.75	block08	0
f04	Incogruent	r0.6	block08	0
f04	Partially Congruent	r0.6	block08	1
f04	Partially Congruent	r0.75	block08	0
f04	Incogruent	r0.75	block08	0
f04	Incogruent	r0.6	block08	1
f04	Partially Congruent	r0.6	block08	0
f04	Fully Congruent	r0.6	block08	0
f04	Fully Congruent	r0.75	block08	1
f04	Partially Congruent	r0.6	block08	0
f04	Fully Congruent	r0.6	block08	0
f04	Fully Congruent	r0.6	block08	0
f04	Partially Congruent	r0.75	block08	1
f04	Partially Congruent	r0.75	block08	0
f04	Partially Congruent	r0.75	block08	1
f04	Partially Congruent	r0.6	block08	0
f04	Partially Congruent	r0.75	block08	1
f04	Fully Congruent	r0.6	block08	0
f04	Incogruent	r0.75	block08	0
f04	Incogruent	r0.6	block08	1
f04	Partially Congruent	r0.6	block08	1
f04	Fully Congruent	r0.6	block08	0
f04	Incogruent	r0.6	block08	1
f04	Fully Congruent	r0.75	block08	1
f04	Partially Congruent	r0.75	block08	0
f04	Fully Congruent	r0.75	block08	0
f04	Incogruent	r0.6	block08	1
f04	Fully Congruent	r0.75	block08	0
f04	Incogruent	r0.6	block08	1

f04	Fully Congruent	r0.75	block08	1
f04	Partially Congruent	r0.75	block08	1
f04	Incogruent	r0.6	block08	0
f04	Fully Congruent	r0.75	block08	1
f04	Incogruent	r0.6	block08	0
f04	Incogruent	r0.6	block08	0
f04	Fully Congruent	r0.75	block08	1
f04	Fully Congruent	r0.6	block08	0
f04	Incogruent	r0.75	block08	1
f04	Incogruent	r0.75	block08	1
f04	Partially Congruent	r0.6	block08	1
f04	Partially Congruent	r0.75	block08	1
f04	Fully Congruent	r0.75	block08	1
f04	Partially Congruent	r0.6	block08	1
f04	Fully Congruent	r0.75	block09	1
f04	Fully Congruent	r0.6	block09	0
f04	Fully Congruent	r0.75	block09	1
f04	Incogruent	r0.6	block09	0
f04	Incogruent	r0.75	block09	0
f04	Partially Congruent	r0.6	block09	1
f04	Incogruent	r0.6	block09	0
f04	Partially Congruent	r0.75	block09	1
f04	Partially Congruent	r0.75	block09	0
f04	Fully Congruent	r0.75	block09	1
f04	Incogruent	r0.75	block09	0
f04	Partially Congruent	r0.6	block09	1
f04	Fully Congruent	r0.75	block09	1
f04	Incogruent	r0.75	block09	0
f04	Fully Congruent	r0.75	block09	0
f04	Partially Congruent	r0.75	block09	1
f04	Fully Congruent	r0.6	block09	1
f04	Fully Congruent	r0.75	block09	1
f04	Incogruent	r0.6	block09	1
f04	Fully Congruent	r0.6	block09	1
f04	Partially Congruent	r0.6	block09	1
f04	Partially Congruent	r0.6	block09	1
f04	Partially Congruent	r0.75	block09	0
f04	Partially Congruent	r0.75	block09	0
f04	Incogruent	r0.75	block09	1
f04	Fully Congruent	r0.75	block09	0
f04	Partially Congruent	r0.75	block09	0
f04	Incogruent	r0.6	block09	1
f04	Partially Congruent	r0.75	block09	0
f04	Partially Congruent	r0.75	block09	0
f04	Fully Congruent	r0.75	block09	0

f04	Partially Congruent	r0.6	block09	0
f04	Incogruent	r0.75	block09	0
f04	Fully Congruent	r0.75	block09	1
f04	Fully Congruent	r0.6	block09	1
f04	Fully Congruent	r0.6	block09	0
f04	Incogruent	r0.75	block09	1
f04	Fully Congruent	r0.75	block09	0
f04	Incogruent	r0.75	block09	0
f04	Fully Congruent	r0.75	block09	0
f04	Fully Congruent	r0.75	block09	1
f04	Partially Congruent	r0.6	block09	1
f04	Partially Congruent	r0.6	block09	1
f04	Incogruent	r0.75	block09	1
f04	Fully Congruent	r0.75	block09	1
f04	Incogruent	r0.6	block09	0
f04	Incogruent	r0.75	block09	0
f04	Fully Congruent	r0.75	block09	1
f04	Incogruent	r0.6	block09	1
f04	Fully Congruent	r0.75	block09	0
m02	Fully Congruent	r0.6	block01	0
m02	Fully Congruent	r0.75	block01	1
m02	Fully Congruent	r0.75	block01	1
m02	Fully Congruent	r0.6	block01	1
m02	Fully Congruent	r0.75	block01	0
m02	Fully Congruent	r0.75	block01	1
m02	Fully Congruent	r0.75	block01	0
m02	Fully Congruent	r0.75	block01	0
m02	Incogruent	r0.75	block01	1
m02	Incogruent	r0.6	block01	0
m02	Fully Congruent	r0.75	block01	1
m02	Partially Congruent	r0.75	block01	0
m02	Incogruent	r0.75	block01	1
m02	Partially Congruent	r0.75	block01	0
m02	Incogruent	r0.6	block01	1
m02	Partially Congruent	r0.6	block01	1
m02	Incogruent	r0.75	block01	0
m02	Partially Congruent	r0.75	block01	1
m02	Fully Congruent	r0.75	block01	1
m02	Incogruent	r0.6	block01	1
m02	Fully Congruent	r0.75	block01	0
m02	Fully Congruent	r0.6	block01	1
m02	Fully Congruent	r0.6	block01	0
m02	Incogruent	r0.75	block01	0
m02	Partially Congruent	r0.6	block01	1
m02	Incogruent	r0.75	block01	1

m02	Incogruent	r0.75	block01	0
m02	Partially Congruent	r0.6	block01	1
m02	Fully Congruent	r0.75	block01	1
m02	Incogruent	r0.6	block01	1
m02	Incogruent	r0.75	block01	1
m02	Fully Congruent	r0.75	block01	0
m02	Fully Congruent	r0.6	block01	0
m02	Fully Congruent	r0.75	block01	1
m02	Incogruent	r0.75	block01	0
m02	Fully Congruent	r0.6	block01	1
m02	Fully Congruent	r0.75	block01	0
m02	Incogruent	r0.75	block01	1
m02	Partially Congruent	r0.75	block01	1
m02	Fully Congruent	r0.75	block01	0
m02	Incogruent	r0.75	block01	1
m02	Partially Congruent	r0.75	block01	0
m02	Incogruent	r0.75	block01	1
m02	Partially Congruent	r0.6	block01	0
m02	Partially Congruent	r0.6	block01	1
m02	Fully Congruent	r0.6	block01	0
m02	Partially Congruent	r0.75	block01	1
m02	Fully Congruent	r0.75	block01	0
m02	Fully Congruent	r0.75	block01	0
m02	Partially Congruent	r0.75	block01	1
m02	Fully Congruent	r0.6	block02	1
m02	Fully Congruent	r0.6	block02	0
m02	Incogruent	r0.75	block02	0
m02	Fully Congruent	r0.75	block02	1
m02	Partially Congruent	r0.6	block02	1
m02	Fully Congruent	r0.75	block02	0
m02	Incogruent	r0.75	block02	1
m02	Fully Congruent	r0.75	block02	0
m02	Fully Congruent	r0.75	block02	0
m02	Incogruent	r0.6	block02	1
m02	Partially Congruent	r0.6	block02	0
m02	Incogruent	r0.6	block02	0
m02	Incogruent	r0.6	block02	1
m02	Fully Congruent	r0.6	block02	0
m02	Partially Congruent	r0.75	block02	1
m02	Partially Congruent	r0.6	block02	0
m02	Fully Congruent	r0.75	block02	1
m02	Incogruent	r0.6	block02	1
m02	Fully Congruent	r0.6	block02	1
m02	Incogruent	r0.75	block02	1
m02	Partially Congruent	r0.6	block02	0

m02	Fully Congruent	r0.6	block02	0
m02	Incogruent	r0.75	block02	1
m02	Partially Congruent	r0.75	block02	1
m02	Fully Congruent	r0.75	block02	1
m02	Partially Congruent	r0.6	block02	1
m02	Fully Congruent	r0.75	block02	1
m02	Partially Congruent	r0.6	block02	0
m02	Fully Congruent	r0.75	block02	1
m02	Incogruent	r0.6	block02	1
m02	Partially Congruent	r0.75	block02	0
m02	Fully Congruent	r0.75	block02	0
m02	Fully Congruent	r0.6	block02	1
m02	Fully Congruent	r0.6	block02	1
m02	Fully Congruent	r0.75	block02	1
m02	Fully Congruent	r0.75	block02	1
m02	Partially Congruent	r0.75	block02	1
m02	Partially Congruent	r0.75	block02	0
m02	Fully Congruent	r0.6	block02	1
m02	Incogruent	r0.75	block02	0
m02	Incogruent	r0.6	block02	1
m02	Fully Congruent	r0.75	block02	0
m02	Partially Congruent	r0.6	block02	1
m02	Incogruent	r0.75	block02	1
m02	Fully Congruent	r0.75	block02	1
m02	Fully Congruent	r0.75	block02	0
m02	Fully Congruent	r0.75	block02	1
m02	Incogruent	r0.75	block02	0
m02	Fully Congruent	r0.75	block02	1
m02	Incogruent	r0.6	block02	1
m02	Incogruent	r0.75	block03	1
m02	Incogruent	r0.75	block03	0
m02	Incogruent	r0.75	block03	1
m02	Incogruent	r0.75	block03	1
m02	Fully Congruent	r0.75	block03	1
m02	Fully Congruent	r0.75	block03	0
m02	Fully Congruent	r0.6	block03	1
m02	Fully Congruent	r0.75	block03	1
m02	Partially Congruent	r0.6	block03	1
m02	Incogruent	r0.75	block03	0
m02	Incogruent	r0.6	block03	1
m02	Partially Congruent	r0.75	block03	0
m02	Fully Congruent	r0.6	block03	0
m02	Incogruent	r0.75	block03	1
m02	Incogruent	r0.6	block03	0
m02	Partially Congruent	r0.6	block03	0

m02	Fully Congruent	r0.75	block03	1
m02	Fully Congruent	r0.75	block03	1
m02	Partially Congruent	r0.6	block03	0
m02	Incogruent	r0.75	block03	0
m02	Partially Congruent	r0.75	block03	0
m02	Partially Congruent	r0.75	block03	0
m02	Fully Congruent	r0.6	block03	0
m02	Partially Congruent	r0.75	block03	0
m02	Fully Congruent	r0.6	block03	1
m02	Fully Congruent	r0.75	block03	0
m02	Fully Congruent	r0.6	block03	1
m02	Partially Congruent	r0.75	block03	1
m02	Partially Congruent	r0.6	block03	1
m02	Fully Congruent	r0.75	block03	0
m02	Fully Congruent	r0.6	block03	1
m02	Fully Congruent	r0.75	block03	0
m02	Incogruent	r0.75	block03	0
m02	Incogruent	r0.6	block03	1
m02	Fully Congruent	r0.6	block03	1
m02	Incogruent	r0.75	block03	0
m02	Partially Congruent	r0.6	block03	0
m02	Incogruent	r0.75	block03	0
m02	Incogruent	r0.6	block03	1
m02	Partially Congruent	r0.6	block03	1
m02	Incogruent	r0.6	block03	0
m02	Partially Congruent	r0.75	block03	0
m02	Fully Congruent	r0.75	block03	1
m02	Fully Congruent	r0.75	block03	0
m02	Fully Congruent	r0.75	block03	1
m02	Partially Congruent	r0.6	block03	0
m02	Fully Congruent	r0.6	block03	1
m02	Incogruent	r0.75	block03	1
m02	Partially Congruent	r0.6	block03	1
m02	Incogruent	r0.75	block03	0
m02	Incogruent	r0.6	block04	0
m02	Incogruent	r0.75	block04	1
m02	Partially Congruent	r0.6	block04	1
m02	Fully Congruent	r0.75	block04	1
m02	Fully Congruent	r0.75	block04	0
m02	Incogruent	r0.6	block04	0
m02	Incogruent	r0.75	block04	0
m02	Partially Congruent	r0.6	block04	1
m02	Incogruent	r0.6	block04	1
m02	Incogruent	r0.6	block04	0
m02	Partially Congruent	r0.75	block04	0

m02	Fully Congruent	r0.75	block04	0
m02	Fully Congruent	r0.75	block04	1
m02	Fully Congruent	r0.75	block04	1
m02	Incogruent	r0.6	block04	1
m02	Incogruent	r0.75	block04	1
m02	Fully Congruent	r0.75	block04	1
m02	Fully Congruent	r0.6	block04	0
m02	Partially Congruent	r0.6	block04	1
m02	Fully Congruent	r0.75	block04	1
m02	Incogruent	r0.6	block04	1
m02	Partially Congruent	r0.75	block04	1
m02	Partially Congruent	r0.75	block04	0
m02	Fully Congruent	r0.75	block04	1
m02	Partially Congruent	r0.6	block04	0
m02	Incogruent	r0.75	block04	1
m02	Fully Congruent	r0.6	block04	1
m02	Incogruent	r0.6	block04	1
m02	Incogruent	r0.75	block04	1
m02	Fully Congruent	r0.75	block04	0
m02	Partially Congruent	r0.75	block04	1
m02	Fully Congruent	r0.75	block04	1
m02	Fully Congruent	r0.6	block04	0
m02	Fully Congruent	r0.75	block04	1
m02	Incogruent	r0.6	block04	1
m02	Incogruent	r0.75	block04	0
m02	Partially Congruent	r0.75	block04	1
m02	Partially Congruent	r0.6	block04	0
m02	Partially Congruent	r0.75	block04	1
m02	Incogruent	r0.75	block04	1
m02	Incogruent	r0.6	block04	1
m02	Fully Congruent	r0.6	block04	1
m02	Partially Congruent	r0.75	block04	1
m02	Incogruent	r0.6	block04	0
m02	Partially Congruent	r0.75	block04	0
m02	Fully Congruent	r0.75	block04	1
m02	Fully Congruent	r0.75	block04	1
m02	Fully Congruent	r0.75	block04	0
m02	Partially Congruent	r0.75	block04	0
m02	Partially Congruent	r0.6	block04	1
m02	Partially Congruent	r0.75	block05	0
m02	Incogruent	r0.6	block05	0
m02	Incogruent	r0.6	block05	1
m02	Partially Congruent	r0.6	block05	0
m02	Partially Congruent	r0.75	block05	0
m02	Incogruent	r0.6	block05	0

m02	Partially Congruent	r0.6	block05	1
m02	Fully Congruent	r0.6	block05	1
m02	Fully Congruent	r0.75	block05	0
m02	Incogruent	r0.6	block05	0
m02	Partially Congruent	r0.6	block05	1
m02	Partially Congruent	r0.6	block05	1
m02	Partially Congruent	r0.6	block05	0
m02	Fully Congruent	r0.75	block05	1
m02	Incogruent	r0.6	block05	1
m02	Incogruent	r0.6	block05	1
m02	Partially Congruent	r0.6	block05	1
m02	Fully Congruent	r0.75	block05	0
m02	Incogruent	r0.6	block05	0
m02	Incogruent	r0.6	block05	0
m02	Fully Congruent	r0.75	block05	0
m02	Fully Congruent	r0.6	block05	1
m02	Incogruent	r0.6	block05	0
m02	Incogruent	r0.75	block05	1
m02	Incogruent	r0.75	block05	1
m02	Fully Congruent	r0.75	block05	0
m02	Fully Congruent	r0.6	block05	1
m02	Fully Congruent	r0.6	block05	0
m02	Partially Congruent	r0.6	block05	0
m02	Incogruent	r0.75	block05	1
m02	Fully Congruent	r0.6	block05	0
m02	Fully Congruent	r0.75	block05	0
m02	Fully Congruent	r0.75	block05	0
m02	Incogruent	r0.6	block05	1
m02	Fully Congruent	r0.75	block05	0
m02	Partially Congruent	r0.6	block05	0
m02	Partially Congruent	r0.75	block05	0
m02	Partially Congruent	r0.75	block05	0
m02	Incogruent	r0.75	block05	0
m02	Fully Congruent	r0.75	block05	1
m02	Incogruent	r0.75	block05	0
m02	Incogruent	r0.75	block05	0
m02	Incogruent	r0.75	block05	0
m02	Fully Congruent	r0.6	block05	1
m02	Fully Congruent	r0.6	block05	1
m02	Fully Congruent	r0.75	block05	1
m02	Partially Congruent	r0.6	block05	1
m02	Incogruent	r0.75	block05	0
m02	Incogruent	r0.6	block05	0
m02	Fully Congruent	r0.75	block05	1
m02	Partially Congruent	r0.6	block06	0

m02	Fully Congruent	r0.75	block06	1
m02	Incogruent	r0.75	block06	1
m02	Incogruent	r0.6	block06	0
m02	Fully Congruent	r0.75	block06	1
m02	Fully Congruent	r0.75	block06	0
m02	Incogruent	r0.6	block06	0
m02	Incogruent	r0.6	block06	1
m02	Fully Congruent	r0.75	block06	1
m02	Incogruent	r0.6	block06	1
m02	Fully Congruent	r0.75	block06	1
m02	Partially Congruent	r0.75	block06	0
m02	Incogruent	r0.6	block06	1
m02	Incogruent	r0.6	block06	1
m02	Partially Congruent	r0.6	block06	0
m02	Fully Congruent	r0.75	block06	1
m02	Partially Congruent	r0.6	block06	0
m02	Partially Congruent	r0.6	block06	1
m02	Partially Congruent	r0.75	block06	1
m02	Partially Congruent	r0.6	block06	1
m02	Partially Congruent	r0.6	block06	1
m02	Fully Congruent	r0.75	block06	0
m02	Incogruent	r0.6	block06	0
m02	Fully Congruent	r0.75	block06	1
m02	Partially Congruent	r0.6	block06	0
m02	Incogruent	r0.6	block06	0
m02	Incogruent	r0.6	block06	1
m02	Partially Congruent	r0.6	block06	1
m02	Partially Congruent	r0.75	block06	0
m02	Fully Congruent	r0.75	block06	0
m02	Partially Congruent	r0.6	block06	1
m02	Partially Congruent	r0.75	block06	1
m02	Partially Congruent	r0.75	block06	1
m02	Incogruent	r0.6	block06	1
m02	Fully Congruent	r0.75	block06	0
m02	Incogruent	r0.6	block06	0
m02	Partially Congruent	r0.6	block06	1
m02	Incogruent	r0.6	block06	1
m02	Incogruent	r0.6	block06	0
m02	Fully Congruent	r0.75	block06	0
m02	Fully Congruent	r0.6	block06	1
m02	Incogruent	r0.6	block06	0
m02	Partially Congruent	r0.6	block06	0
m02	Fully Congruent	r0.75	block06	1
m02	Partially Congruent	r0.75	block06	1
m02	Fully Congruent	r0.75	block06	0

m02	Incogruent	r0.6	block06	1
m02	Fully Congruent	r0.6	block06	1
m02	Incogruent	r0.75	block06	0
m02	Partially Congruent	r0.75	block06	0
m02	Fully Congruent	r0.6	block07	0
m02	Incogruent	r0.6	block07	0
m02	Partially Congruent	r0.75	block07	0
m02	Incogruent	r0.6	block07	0
m02	Partially Congruent	r0.75	block07	0
m02	Incogruent	r0.6	block07	0
m02	Partially Congruent	r0.75	block07	1
m02	Incogruent	r0.6	block07	1
m02	Fully Congruent	r0.75	block07	1
m02	Partially Congruent	r0.75	block07	1
m02	Partially Congruent	r0.75	block07	1
m02	Partially Congruent	r0.75	block07	1
m02	Partially Congruent	r0.75	block07	1
m02	Incogruent	r0.6	block07	1
m02	Incogruent	r0.6	block07	1
m02	Incogruent	r0.6	block07	1
m02	Incogruent	r0.75	block07	1
m02	Fully Congruent	r0.75	block07	0
m02	Fully Congruent	r0.75	block07	0
m02	Fully Congruent	r0.75	block07	1
m02	Partially Congruent	r0.6	block07	1
m02	Incogruent	r0.6	block07	0
m02	Partially Congruent	r0.6	block07	1
m02	Incogruent	r0.75	block07	1
m02	Partially Congruent	r0.75	block07	0
m02	Partially Congruent	r0.6	block07	0
m02	Partially Congruent	r0.6	block07	0
m02	Partially Congruent	r0.6	block07	1
m02	Incogruent	r0.75	block07	1
m02	Fully Congruent	r0.75	block07	1
m02	Incogruent	r0.75	block07	1
m02	Partially Congruent	r0.6	block07	1
m02	Incogruent	r0.75	block07	1
m02	Partially Congruent	r0.75	block07	1
m02	Fully Congruent	r0.6	block07	1
m02	Fully Congruent	r0.75	block07	0
m02	Incogruent	r0.6	block07	1
m02	Incogruent	r0.6	block07	1
m02	Incogruent	r0.6	block07	0
m02	Fully Congruent	r0.75	block07	1
m02	Partially Congruent	r0.75	block07	0

m02	Partially Congruent	r0.75	block07	0
m02	Partially Congruent	r0.6	block07	0
m02	Partially Congruent	r0.6	block07	1
m02	Fully Congruent	r0.6	block07	1
m02	Incogruent	r0.6	block07	1
m02	Partially Congruent	r0.75	block07	0
m02	Partially Congruent	r0.75	block07	1
m02	Partially Congruent	r0.6	block07	0
m02	Partially Congruent	r0.6	block07	1
m02	Fully Congruent	r0.6	block08	1
m02	Incogruent	r0.75	block08	1
m02	Fully Congruent	r0.6	block08	1
m02	Fully Congruent	r0.75	block08	0
m02	Incogruent	r0.75	block08	1
m02	Incogruent	r0.6	block08	1
m02	Incogruent	r0.75	block08	0
m02	Fully Congruent	r0.75	block08	1
m02	Fully Congruent	r0.6	block08	0
m02	Partially Congruent	r0.75	block08	0
m02	Fully Congruent	r0.75	block08	1
m02	Incogruent	r0.6	block08	0
m02	Partially Congruent	r0.75	block08	1
m02	Incogruent	r0.6	block08	0
m02	Incogruent	r0.75	block08	0
m02	Fully Congruent	r0.6	block08	0
m02	Fully Congruent	r0.75	block08	1
m02	Partially Congruent	r0.75	block08	0
m02	Fully Congruent	r0.6	block08	1
m02	Incogruent	r0.75	block08	1
m02	Incogruent	r0.75	block08	1
m02	Partially Congruent	r0.6	block08	0
m02	Fully Congruent	r0.75	block08	1
m02	Incogruent	r0.6	block08	0
m02	Partially Congruent	r0.75	block08	0
m02	Fully Congruent	r0.75	block08	1
m02	Partially Congruent	r0.75	block08	0
m02	Fully Congruent	r0.6	block08	1
m02	Partially Congruent	r0.6	block08	1
m02	Fully Congruent	r0.75	block08	0
m02	Fully Congruent	r0.75	block08	0
m02	Incogruent	r0.6	block08	1
m02	Fully Congruent	r0.75	block08	0
m02	Incogruent	r0.6	block08	0
m02	Incogruent	r0.6	block08	0
m02	Fully Congruent	r0.75	block08	1

m02	Fully Congruent	r0.75	block08	0
m02	Fully Congruent	r0.6	block08	0
m02	Fully Congruent	r0.75	block08	0
m02	Fully Congruent	r0.75	block08	1
m02	Fully Congruent	r0.75	block08	1
m02	Incogruent	r0.75	block08	0
m02	Fully Congruent	r0.75	block08	1
m02	Incogruent	r0.75	block08	1
m02	Partially Congruent	r0.6	block08	1
m02	Incogruent	r0.6	block08	1
m02	Incogruent	r0.6	block08	1
m02	Partially Congruent	r0.6	block08	0
m02	Partially Congruent	r0.6	block08	1
m02	Fully Congruent	r0.75	block08	1
m02	Fully Congruent	r0.6	block09	1
m02	Incogruent	r0.6	block09	0
m02	Fully Congruent	r0.75	block09	0
m02	Incogruent	r0.75	block09	0
m02	Incogruent	r0.6	block09	0
m02	Fully Congruent	r0.75	block09	1
m02	Incogruent	r0.75	block09	1
m02	Incogruent	r0.6	block09	1
m02	Incogruent	r0.6	block09	1
m02	Fully Congruent	r0.75	block09	0
m02	Incogruent	r0.75	block09	1
m02	Incogruent	r0.75	block09	0
m02	Fully Congruent	r0.6	block09	0
m02	Incogruent	r0.6	block09	0
m02	Incogruent	r0.6	block09	1
m02	Fully Congruent	r0.6	block09	0
m02	Partially Congruent	r0.6	block09	0
m02	Fully Congruent	r0.6	block09	1
m02	Partially Congruent	r0.75	block09	0
m02	Incogruent	r0.6	block09	1
m02	Fully Congruent	r0.6	block09	1
m02	Partially Congruent	r0.6	block09	1
m02	Partially Congruent	r0.6	block09	1
m02	Incogruent	r0.6	block09	1
m02	Fully Congruent	r0.75	block09	0
m02	Fully Congruent	r0.75	block09	0
m02	Incogruent	r0.75	block09	1
m02	Fully Congruent	r0.6	block09	0
m02	Partially Congruent	r0.6	block09	0
m02	Partially Congruent	r0.6	block09	1
m02	Incogruent	r0.75	block09	1

m02	Incogruent	r0.75	block09	1
m02	Partially Congruent	r0.75	block09	1
m02	Fully Congruent	r0.75	block09	0
m02	Partially Congruent	r0.6	block09	1
m02	Incogruent	r0.75	block09	0
m02	Fully Congruent	r0.75	block09	0
m02	Fully Congruent	r0.75	block09	1
m02	Fully Congruent	r0.75	block09	1
m02	Incogruent	r0.75	block09	1
m02	Incogruent	r0.75	block09	1
m02	Incogruent	r0.75	block09	0
m02	Incogruent	r0.75	block09	0
m02	Partially Congruent	r0.6	block09	1
m02	Fully Congruent	r0.75	block09	1
m02	Partially Congruent	r0.75	block09	0
m02	Incogruent	r0.75	block09	1
m02	Incogruent	r0.6	block09	1
m02	Partially Congruent	r0.6	block09	1
m02	Partially Congruent	r0.6	block09	1
m02	Incogruent	r0.75	block10	0
m02	Partially Congruent	r0.6	block10	1
m02	Partially Congruent	r0.6	block10	1
m02	Incogruent	r0.6	block10	1
m02	Incogruent	r0.75	block10	1
m02	Partially Congruent	r0.6	block10	0
m02	Partially Congruent	r0.6	block10	1
m02	Fully Congruent	r0.6	block10	1
m02	Incogruent	r0.6	block10	1
m02	Fully Congruent	r0.75	block10	1
m02	Incogruent	r0.6	block10	0
m02	Partially Congruent	r0.75	block10	1
m02	Fully Congruent	r0.75	block10	1
m02	Fully Congruent	r0.75	block10	1
m02	Partially Congruent	r0.75	block10	0
m02	Partially Congruent	r0.75	block10	0
m02	Partially Congruent	r0.6	block10	1
m02	Fully Congruent	r0.75	block10	0
m02	Incogruent	r0.6	block10	1
m02	Incogruent	r0.6	block10	0
m02	Incogruent	r0.6	block10	1
m02	Fully Congruent	r0.6	block10	0
m02	Fully Congruent	r0.6	block10	1
m02	Partially Congruent	r0.6	block10	0
m02	Partially Congruent	r0.6	block10	1
m02	Partially Congruent	r0.6	block10	1

m02	Fully Congruent	r0.75	block10	1
m02	Fully Congruent	r0.75	block10	0
m02	Incogruent	r0.6	block10	1
m02	Fully Congruent	r0.75	block10	1
m02	Partially Congruent	r0.6	block10	1
m02	Fully Congruent	r0.75	block10	1
m02	Incogruent	r0.6	block10	1
m02	Incogruent	r0.6	block10	1
m02	Partially Congruent	r0.75	block10	1
m02	Fully Congruent	r0.75	block10	0
m02	Incogruent	r0.6	block10	1
m02	Partially Congruent	r0.75	block10	1
m02	Incogruent	r0.6	block10	1
m02	Fully Congruent	r0.75	block10	1
m02	Incogruent	r0.6	block10	1
m02	Incogruent	r0.75	block10	1
m02	Incogruent	r0.75	block10	0
m02	Incogruent	r0.75	block10	1
m02	Partially Congruent	r0.6	block10	0
m02	Fully Congruent	r0.75	block10	0
m02	Fully Congruent	r0.75	block10	1
m02	Fully Congruent	r0.6	block10	0
m02	Partially Congruent	r0.6	block10	1
m02	Partially Congruent	r0.6	block10	1
m03	Fully Congruent	r0.6	block01	0
m03	Incogruent	r0.75	block01	1
m03	Incogruent	r0.75	block01	1
m03	Incogruent	r0.75	block01	0
m03	Partially Congruent	r0.75	block01	1
m03	Incogruent	r0.6	block01	0
m03	Fully Congruent	r0.6	block01	1
m03	Incogruent	r0.75	block01	0
m03	Incogruent	r0.75	block01	1
m03	Incogruent	r0.6	block01	1
m03	Partially Congruent	r0.6	block01	1
m03	Incogruent	r0.6	block01	0
m03	Incogruent	r0.75	block01	0
m03	Partially Congruent	r0.6	block01	1
m03	Fully Congruent	r0.75	block01	1
m03	Partially Congruent	r0.6	block01	0
m03	Fully Congruent	r0.6	block01	0
m03	Partially Congruent	r0.6	block01	1
m03	Incogruent	r0.75	block01	1
m03	Incogruent	r0.6	block01	0
m03	Fully Congruent	r0.75	block01	0

m03	Incogruent	r0.6	block01	1
m03	Incogruent	r0.75	block01	0
m03	Fully Congruent	r0.75	block01	0
m03	Fully Congruent	r0.75	block01	1
m03	Incogruent	r0.75	block01	1
m03	Partially Congruent	r0.6	block01	1
m03	Fully Congruent	r0.75	block01	1
m03	Partially Congruent	r0.75	block01	1
m03	Incogruent	r0.6	block01	0
m03	Partially Congruent	r0.6	block01	1
m03	Incogruent	r0.6	block01	0
m03	Partially Congruent	r0.6	block01	0
m03	Incogruent	r0.6	block01	0
m03	Partially Congruent	r0.75	block01	1
m03	Incogruent	r0.75	block01	1
m03	Fully Congruent	r0.75	block01	0
m03	Partially Congruent	r0.75	block01	1
m03	Fully Congruent	r0.75	block01	0
m03	Fully Congruent	r0.6	block01	0
m03	Partially Congruent	r0.75	block01	0
m03	Partially Congruent	r0.6	block01	0
m03	Incogruent	r0.6	block01	0
m03	Incogruent	r0.6	block01	1
m03	Partially Congruent	r0.6	block01	1
m03	Incogruent	r0.75	block01	0
m03	Incogruent	r0.6	block01	0
m03	Incogruent	r0.6	block01	0
m03	Partially Congruent	r0.6	block01	0
m03	Partially Congruent	r0.6	block01	0
m03	Incogruent	r0.75	block02	1
m03	Incogruent	r0.6	block02	0
m03	Partially Congruent	r0.6	block02	1
m03	Fully Congruent	r0.75	block02	1
m03	Fully Congruent	r0.75	block02	1
m03	Partially Congruent	r0.6	block02	0
m03	Incogruent	r0.6	block02	1
m03	Incogruent	r0.6	block02	0
m03	Partially Congruent	r0.6	block02	0
m03	Fully Congruent	r0.75	block02	0
m03	Partially Congruent	r0.6	block02	0
m03	Fully Congruent	r0.75	block02	0
m03	Partially Congruent	r0.6	block02	1
m03	Incogruent	r0.75	block02	1
m03	Fully Congruent	r0.75	block02	1
m03	Fully Congruent	r0.75	block02	1

m03	Fully Congruent	r0.75	block02	1
m03	Fully Congruent	r0.6	block02	0
m03	Incogruent	r0.6	block02	0
m03	Fully Congruent	r0.75	block02	1
m03	Fully Congruent	r0.75	block02	0
m03	Partially Congruent	r0.6	block02	1
m03	Fully Congruent	r0.75	block02	1
m03	Fully Congruent	r0.6	block02	1
m03	Partially Congruent	r0.6	block02	1
m03	Partially Congruent	r0.6	block02	0
m03	Fully Congruent	r0.75	block02	1
m03	Fully Congruent	r0.6	block02	1
m03	Partially Congruent	r0.6	block02	0
m03	Fully Congruent	r0.6	block02	0
m03	Incogruent	r0.75	block02	0
m03	Incogruent	r0.75	block02	1
m03	Incogruent	r0.6	block02	0
m03	Fully Congruent	r0.6	block02	1
m03	Incogruent	r0.75	block02	1
m03	Incogruent	r0.6	block02	0
m03	Fully Congruent	r0.6	block02	1
m03	Incogruent	r0.75	block02	0
m03	Partially Congruent	r0.6	block02	0
m03	Incogruent	r0.6	block02	1
m03	Partially Congruent	r0.75	block02	0
m03	Incogruent	r0.6	block02	0
m03	Fully Congruent	r0.75	block02	1
m03	Fully Congruent	r0.75	block02	0
m03	Partially Congruent	r0.6	block02	0
m03	Incogruent	r0.75	block02	0
m03	Partially Congruent	r0.6	block02	0
m03	Fully Congruent	r0.75	block02	1
m03	Partially Congruent	r0.6	block02	1
m03	Fully Congruent	r0.6	block02	0
m03	Partially Congruent	r0.6	block03	1
m03	Fully Congruent	r0.75	block03	1
m03	Incogruent	r0.75	block03	0
m03	Incogruent	r0.6	block03	0
m03	Fully Congruent	r0.75	block03	1
m03	Partially Congruent	r0.75	block03	0
m03	Partially Congruent	r0.75	block03	1
m03	Incogruent	r0.75	block03	1
m03	Incogruent	r0.75	block03	0
m03	Partially Congruent	r0.75	block03	1
m03	Incogruent	r0.6	block03	1

m03	Partially Congruent	r0.75	block03	0
m03	Fully Congruent	r0.75	block03	1
m03	Partially Congruent	r0.6	block03	0
m03	Partially Congruent	r0.6	block03	0
m03	Partially Congruent	r0.6	block03	0
m03	Fully Congruent	r0.75	block03	1
m03	Incogruent	r0.6	block03	1
m03	Fully Congruent	r0.6	block03	1
m03	Fully Congruent	r0.6	block03	0
m03	Partially Congruent	r0.75	block03	0
m03	Fully Congruent	r0.6	block03	1
m03	Partially Congruent	r0.6	block03	1
m03	Incogruent	r0.75	block03	0
m03	Fully Congruent	r0.6	block03	1
m03	Incogruent	r0.75	block03	0
m03	Fully Congruent	r0.75	block03	0
m03	Partially Congruent	r0.75	block03	1
m03	Fully Congruent	r0.6	block03	1
m03	Fully Congruent	r0.6	block03	1
m03	Partially Congruent	r0.6	block03	0
m03	Partially Congruent	r0.6	block03	0
m03	Partially Congruent	r0.75	block03	1
m03	Partially Congruent	r0.75	block03	0
m03	Incogruent	r0.6	block03	1
m03	Partially Congruent	r0.6	block03	1
m03	Partially Congruent	r0.6	block03	0
m03	Incogruent	r0.6	block03	1
m03	Fully Congruent	r0.75	block03	0
m03	Fully Congruent	r0.6	block03	1
m03	Incogruent	r0.6	block03	1
m03	Fully Congruent	r0.6	block03	0
m03	Partially Congruent	r0.6	block03	1
m03	Partially Congruent	r0.6	block03	0
m03	Partially Congruent	r0.75	block03	1
m03	Fully Congruent	r0.75	block03	0
m03	Incogruent	r0.6	block03	0
m03	Partially Congruent	r0.6	block03	0
m03	Fully Congruent	r0.75	block03	1
m03	Fully Congruent	r0.75	block03	1
m03	Fully Congruent	r0.75	block04	1
m03	Incogruent	r0.6	block04	1
m03	Incogruent	r0.75	block04	0
m03	Fully Congruent	r0.6	block04	1
m03	Partially Congruent	r0.6	block04	0
m03	Partially Congruent	r0.75	block04	1

m03	Fully Congruent	r0.75	block04	1
m03	Incogruent	r0.6	block04	1
m03	Incogruent	r0.6	block04	0
m03	Incogruent	r0.75	block04	0
m03	Fully Congruent	r0.6	block04	1
m03	Incogruent	r0.6	block04	1
m03	Incogruent	r0.6	block04	1
m03	Fully Congruent	r0.75	block04	1
m03	Partially Congruent	r0.6	block04	1
m03	Partially Congruent	r0.6	block04	0
m03	Partially Congruent	r0.75	block04	1
m03	Fully Congruent	r0.75	block04	1
m03	Incogruent	r0.6	block04	1
m03	Fully Congruent	r0.75	block04	1
m03	Fully Congruent	r0.75	block04	0
m03	Incogruent	r0.6	block04	1
m03	Incogruent	r0.75	block04	0
m03	Partially Congruent	r0.75	block04	1
m03	Incogruent	r0.75	block04	1
m03	Fully Congruent	r0.6	block04	1
m03	Partially Congruent	r0.75	block04	1
m03	Incogruent	r0.6	block04	1
m03	Fully Congruent	r0.75	block04	1
m03	Partially Congruent	r0.75	block04	0
m03	Fully Congruent	r0.6	block04	0
m03	Partially Congruent	r0.75	block04	1
m03	Incogruent	r0.75	block04	0
m03	Incogruent	r0.6	block04	0
m03	Fully Congruent	r0.6	block04	1
m03	Incogruent	r0.75	block04	1
m03	Partially Congruent	r0.6	block04	1
m03	Incogruent	r0.75	block04	1
m03	Incogruent	r0.75	block04	1
m03	Partially Congruent	r0.75	block04	1
m03	Fully Congruent	r0.75	block04	0
m03	Incogruent	r0.75	block04	0
m03	Partially Congruent	r0.75	block04	0
m03	Partially Congruent	r0.6	block04	0
m03	Fully Congruent	r0.75	block04	1
m03	Incogruent	r0.75	block04	0
m03	Partially Congruent	r0.6	block04	1
m03	Incogruent	r0.75	block04	1
m03	Fully Congruent	r0.75	block04	1
m03	Fully Congruent	r0.75	block04	1
m03	Partially Congruent	r0.75	block05	0

m03	Partially Congruent	r0.75	block05	0
m03	Fully Congruent	r0.75	block05	1
m03	Fully Congruent	r0.75	block05	0
m03	Partially Congruent	r0.6	block05	1
m03	Partially Congruent	r0.6	block05	0
m03	Fully Congruent	r0.6	block05	1
m03	Fully Congruent	r0.75	block05	0
m03	Fully Congruent	r0.75	block05	1
m03	Partially Congruent	r0.6	block05	1
m03	Incogruent	r0.6	block05	1
m03	Partially Congruent	r0.6	block05	0
m03	Partially Congruent	r0.75	block05	0
m03	Partially Congruent	r0.75	block05	0
m03	Partially Congruent	r0.75	block05	0
m03	Incogruent	r0.75	block05	0
m03	Partially Congruent	r0.75	block05	0
m03	Fully Congruent	r0.75	block05	1
m03	Fully Congruent	r0.75	block05	1
m03	Partially Congruent	r0.6	block05	1
m03	Fully Congruent	r0.6	block05	1
m03	Incogruent	r0.6	block05	0
m03	Partially Congruent	r0.75	block05	0
m03	Partially Congruent	r0.75	block05	1
m03	Partially Congruent	r0.6	block05	0
m03	Fully Congruent	r0.75	block05	0
m03	Fully Congruent	r0.75	block05	1
m03	Incogruent	r0.6	block05	1
m03	Fully Congruent	r0.6	block05	0
m03	Incogruent	r0.6	block05	0
m03	Incogruent	r0.6	block05	0
m03	Partially Congruent	r0.6	block05	1
m03	Partially Congruent	r0.75	block05	1
m03	Partially Congruent	r0.6	block05	1
m03	Fully Congruent	r0.75	block05	1
m03	Incogruent	r0.75	block05	0
m03	Partially Congruent	r0.6	block05	1
m03	Partially Congruent	r0.6	block05	0
m03	Partially Congruent	r0.75	block05	0
m03	Partially Congruent	r0.75	block05	0
m03	Incogruent	r0.75	block05	1
m03	Incogruent	r0.6	block05	0
m03	Incogruent	r0.75	block05	0
m03	Fully Congruent	r0.75	block05	1
m03	Incogruent	r0.6	block05	0
m03	Fully Congruent	r0.6	block05	0

m03	Fully Congruent	r0.75	block05	1
m03	Partially Congruent	r0.6	block05	1
m03	Incogruent	r0.6	block05	0
m03	Partially Congruent	r0.75	block05	1
m03	Partially Congruent	r0.6	block06	0
m03	Incogruent	r0.6	block06	0
m03	Fully Congruent	r0.6	block06	1
m03	Incogruent	r0.6	block06	1
m03	Fully Congruent	r0.75	block06	1
m03	Incogruent	r0.75	block06	1
m03	Partially Congruent	r0.75	block06	0
m03	Fully Congruent	r0.75	block06	0
m03	Partially Congruent	r0.6	block06	1
m03	Incogruent	r0.75	block06	0
m03	Partially Congruent	r0.75	block06	0
m03	Partially Congruent	r0.75	block06	1
m03	Fully Congruent	r0.75	block06	0
m03	Fully Congruent	r0.75	block06	0
m03	Fully Congruent	r0.75	block06	1
m03	Incogruent	r0.75	block06	0
m03	Incogruent	r0.6	block06	0
m03	Fully Congruent	r0.6	block06	0
m03	Fully Congruent	r0.75	block06	1
m03	Incogruent	r0.6	block06	1
m03	Partially Congruent	r0.75	block06	1
m03	Fully Congruent	r0.75	block06	0
m03	Fully Congruent	r0.6	block06	1
m03	Incogruent	r0.75	block06	0
m03	Incogruent	r0.6	block06	1
m03	Partially Congruent	r0.6	block06	0
m03	Incogruent	r0.6	block06	0
m03	Partially Congruent	r0.75	block06	0
m03	Partially Congruent	r0.75	block06	1
m03	Fully Congruent	r0.75	block06	0
m03	Incogruent	r0.6	block06	0
m03	Partially Congruent	r0.75	block06	0
m03	Incogruent	r0.75	block06	1
m03	Fully Congruent	r0.75	block06	0
m03	Fully Congruent	r0.75	block06	1
m03	Fully Congruent	r0.6	block06	1
m03	Partially Congruent	r0.6	block06	1
m03	Partially Congruent	r0.75	block06	0
m03	Fully Congruent	r0.6	block06	0
m03	Partially Congruent	r0.75	block06	0
m03	Fully Congruent	r0.75	block06	1

m03	Fully Congruent	r0.6	block06	1
m03	Fully Congruent	r0.75	block06	1
m03	Partially Congruent	r0.6	block06	1
m03	Incogruent	r0.75	block06	0
m03	Partially Congruent	r0.6	block06	0
m03	Incogruent	r0.6	block06	1
m03	Partially Congruent	r0.6	block06	1
m03	Fully Congruent	r0.75	block06	1
m03	Incogruent	r0.75	block06	0
m03	Partially Congruent	r0.6	block07	1
m03	Incogruent	r0.6	block07	0
m03	Fully Congruent	r0.75	block07	1
m03	Fully Congruent	r0.75	block07	1
m03	Incogruent	r0.6	block07	1
m03	Fully Congruent	r0.75	block07	1
m03	Fully Congruent	r0.6	block07	0
m03	Partially Congruent	r0.6	block07	0
m03	Incogruent	r0.6	block07	1
m03	Fully Congruent	r0.75	block07	1
m03	Partially Congruent	r0.75	block07	1
m03	Incogruent	r0.6	block07	0
m03	Partially Congruent	r0.75	block07	0
m03	Incogruent	r0.6	block07	0
m03	Partially Congruent	r0.6	block07	1
m03	Incogruent	r0.75	block07	0
m03	Fully Congruent	r0.75	block07	1
m03	Incogruent	r0.6	block07	1
m03	Incogruent	r0.75	block07	0
m03	Partially Congruent	r0.75	block07	0
m03	Incogruent	r0.6	block07	0
m03	Fully Congruent	r0.75	block07	0
m03	Fully Congruent	r0.6	block07	1
m03	Fully Congruent	r0.75	block07	1
m03	Incogruent	r0.75	block07	1
m03	Fully Congruent	r0.75	block07	1
m03	Partially Congruent	r0.75	block07	1
m03	Partially Congruent	r0.75	block07	0
m03	Incogruent	r0.6	block07	1
m03	Partially Congruent	r0.75	block07	1
m03	Fully Congruent	r0.75	block07	1
m03	Partially Congruent	r0.6	block07	0
m03	Partially Congruent	r0.75	block07	1
m03	Incogruent	r0.6	block07	1
m03	Incogruent	r0.75	block07	0
m03	Incogruent	r0.6	block07	1

m03	Partially Congruent	r0.6	block07	0
m03	Incogruent	r0.6	block07	0
m03	Fully Congruent	r0.6	block07	0
m03	Fully Congruent	r0.75	block07	1
m03	Partially Congruent	r0.6	block07	1
m03	Incogruent	r0.75	block07	1
m03	Fully Congruent	r0.75	block07	0
m03	Partially Congruent	r0.6	block07	1
m03	Incogruent	r0.75	block07	1
m03	Partially Congruent	r0.75	block07	1
m03	Fully Congruent	r0.6	block07	1
m03	Incogruent	r0.6	block07	0
m03	Fully Congruent	r0.75	block07	0
m03	Fully Congruent	r0.75	block07	1
m03	Incogruent	r0.6	block08	1
m03	Partially Congruent	r0.6	block08	0
m03	Fully Congruent	r0.75	block08	0
m03	Fully Congruent	r0.6	block08	1
m03	Fully Congruent	r0.75	block08	1
m03	Partially Congruent	r0.6	block08	1
m03	Fully Congruent	r0.6	block08	1
m03	Incogruent	r0.6	block08	0
m03	Partially Congruent	r0.6	block08	1
m03	Fully Congruent	r0.75	block08	1
m03	Fully Congruent	r0.6	block08	0
m03	Incogruent	r0.75	block08	1
m03	Partially Congruent	r0.75	block08	0
m03	Fully Congruent	r0.75	block08	1
m03	Incogruent	r0.6	block08	0
m03	Partially Congruent	r0.6	block08	1
m03	Fully Congruent	r0.75	block08	1
m03	Incogruent	r0.6	block08	1
m03	Partially Congruent	r0.6	block08	1
m03	Fully Congruent	r0.75	block08	1
m03	Partially Congruent	r0.6	block08	1
m03	Incogruent	r0.6	block08	1
m03	Partially Congruent	r0.6	block08	1
m03	Partially Congruent	r0.75	block08	1
m03	Partially Congruent	r0.6	block08	0
m03	Fully Congruent	r0.6	block08	1
m03	Incogruent	r0.75	block08	0
m03	Partially Congruent	r0.75	block08	0
m03	Fully Congruent	r0.75	block08	1
m03	Incogruent	r0.6	block08	1
m03	Fully Congruent	r0.75	block08	1

m03	Incogruent	r0.6	block08	1
m03	Partially Congruent	r0.6	block08	0
m03	Fully Congruent	r0.75	block08	1
m03	Partially Congruent	r0.75	block08	1
m03	Incogruent	r0.6	block08	1
m03	Incogruent	r0.6	block08	1
m03	Incogruent	r0.75	block08	0
m03	Partially Congruent	r0.75	block08	1
m03	Partially Congruent	r0.6	block08	0
m03	Partially Congruent	r0.6	block08	1
m03	Fully Congruent	r0.6	block08	0
m03	Incogruent	r0.75	block08	1
m03	Partially Congruent	r0.6	block08	0
m03	Incogruent	r0.6	block08	0
m03	Fully Congruent	r0.6	block08	1
m03	Incogruent	r0.75	block08	0
m03	Fully Congruent	r0.6	block08	1
m03	Partially Congruent	r0.6	block08	0
m03	Incogruent	r0.6	block08	0
m03	Incogruent	r0.6	block09	0
m03	Partially Congruent	r0.6	block09	1
m03	Incogruent	r0.75	block09	0
m03	Fully Congruent	r0.75	block09	0
m03	Partially Congruent	r0.6	block09	1
m03	Incogruent	r0.75	block09	1
m03	Incogruent	r0.6	block09	1
m03	Partially Congruent	r0.75	block09	0
m03	Partially Congruent	r0.6	block09	0
m03	Incogruent	r0.75	block09	1
m03	Partially Congruent	r0.75	block09	1
m03	Incogruent	r0.75	block09	0
m03	Incogruent	r0.75	block09	1
m03	Partially Congruent	r0.75	block09	0
m03	Fully Congruent	r0.75	block09	1
m03	Fully Congruent	r0.6	block09	1
m03	Incogruent	r0.75	block09	1
m03	Fully Congruent	r0.75	block09	0
m03	Incogruent	r0.6	block09	0
m03	Fully Congruent	r0.75	block09	0
m03	Incogruent	r0.75	block09	0
m03	Fully Congruent	r0.6	block09	0
m03	Partially Congruent	r0.6	block09	1
m03	Incogruent	r0.75	block09	0
m03	Fully Congruent	r0.75	block09	0
m03	Partially Congruent	r0.75	block09	0

m03	Fully Congruent	r0.6	block09	1
m03	Incogruent	r0.6	block09	0
m03	Partially Congruent	r0.75	block09	0
m03	Partially Congruent	r0.6	block09	1
m03	Partially Congruent	r0.6	block09	0
m03	Partially Congruent	r0.6	block09	1
m03	Incogruent	r0.6	block09	1
m03	Fully Congruent	r0.75	block09	0
m03	Fully Congruent	r0.75	block09	0
m03	Partially Congruent	r0.6	block09	1
m03	Fully Congruent	r0.75	block09	0
m03	Partially Congruent	r0.6	block09	1
m03	Fully Congruent	r0.75	block09	0
m03	Incogruent	r0.6	block09	1
m03	Incogruent	r0.6	block09	1
m03	Incogruent	r0.75	block09	0
m03	Incogruent	r0.6	block09	1
m03	Fully Congruent	r0.75	block09	0
m03	Fully Congruent	r0.6	block09	1
m03	Fully Congruent	r0.6	block09	1
m03	Fully Congruent	r0.75	block09	1
m03	Fully Congruent	r0.75	block09	0
m03	Incogruent	r0.75	block09	0
m03	Fully Congruent	r0.75	block09	1
m03	Incogruent	r0.6	block10	0
m03	Partially Congruent	r0.6	block10	0
m03	Fully Congruent	r0.75	block10	1
m03	Partially Congruent	r0.6	block10	1
m03	Incogruent	r0.6	block10	1
m03	Incogruent	r0.75	block10	1
m03	Partially Congruent	r0.75	block10	1
m03	Partially Congruent	r0.75	block10	0
m03	Fully Congruent	r0.75	block10	1
m03	Partially Congruent	r0.6	block10	1
m03	Incogruent	r0.6	block10	1
m03	Incogruent	r0.75	block10	1
m03	Fully Congruent	r0.6	block10	1
m03	Fully Congruent	r0.75	block10	1
m03	Incogruent	r0.75	block10	0
m03	Incogruent	r0.75	block10	1
m03	Fully Congruent	r0.6	block10	1
m03	Partially Congruent	r0.6	block10	0
m03	Fully Congruent	r0.75	block10	1
m03	Partially Congruent	r0.6	block10	1
m03	Incogruent	r0.6	block10	1

m03	Partially Congruent	r0.6	block10	0
m03	Fully Congruent	r0.75	block10	1
m03	Incogruent	r0.6	block10	1
m03	Partially Congruent	r0.6	block10	1
m03	Fully Congruent	r0.6	block10	1
m03	Incogruent	r0.6	block10	0
m03	Fully Congruent	r0.6	block10	1
m03	Incogruent	r0.75	block10	1
m03	Fully Congruent	r0.75	block10	0
m03	Fully Congruent	r0.6	block10	1
m03	Incogruent	r0.75	block10	0
m03	Partially Congruent	r0.6	block10	0
m03	Fully Congruent	r0.75	block10	1
m03	Fully Congruent	r0.6	block10	1
m03	Partially Congruent	r0.75	block10	0
m03	Fully Congruent	r0.75	block10	1
m03	Fully Congruent	r0.6	block10	0
m03	Incogruent	r0.6	block10	0
m03	Partially Congruent	r0.75	block10	1
m03	Fully Congruent	r0.6	block10	1
m03	Fully Congruent	r0.75	block10	1
m03	Incogruent	r0.75	block10	1
m03	Fully Congruent	r0.75	block10	0
m03	Fully Congruent	r0.75	block10	1
m03	Partially Congruent	r0.75	block10	0
m03	Fully Congruent	r0.75	block10	1
m03	Partially Congruent	r0.6	block10	1
m03	Incogruent	r0.6	block10	1
m03	Incogruent	r0.75	block10	0
m04	Fully Congruent	r0.75	block01	1
m04	Fully Congruent	r0.6	block01	0
m04	Partially Congruent	r0.75	block01	0
m04	Partially Congruent	r0.75	block01	0
m04	Fully Congruent	r0.6	block01	1
m04	Incogruent	r0.6	block01	0
m04	Incogruent	r0.6	block01	1
m04	Partially Congruent	r0.75	block01	0
m04	Fully Congruent	r0.6	block01	1
m04	Fully Congruent	r0.6	block01	1
m04	Partially Congruent	r0.6	block01	1
m04	Partially Congruent	r0.6	block01	0
m04	Partially Congruent	r0.75	block01	0
m04	Fully Congruent	r0.75	block01	1
m04	Partially Congruent	r0.6	block01	0
m04	Partially Congruent	r0.75	block01	1

m04	Incogruent	r0.75	block01	1
m04	Partially Congruent	r0.75	block01	0
m04	Partially Congruent	r0.6	block01	0
m04	Incogruent	r0.75	block01	1
m04	Fully Congruent	r0.75	block01	1
m04	Fully Congruent	r0.75	block01	0
m04	Fully Congruent	r0.6	block01	0
m04	Partially Congruent	r0.6	block01	0
m04	Fully Congruent	r0.75	block01	1
m04	Fully Congruent	r0.75	block01	0
m04	Incogruent	r0.75	block01	1
m04	Fully Congruent	r0.6	block01	0
m04	Incogruent	r0.6	block01	1
m04	Partially Congruent	r0.75	block01	1
m04	Partially Congruent	r0.6	block01	1
m04	Fully Congruent	r0.75	block01	0
m04	Incogruent	r0.6	block01	1
m04	Partially Congruent	r0.6	block01	1
m04	Incogruent	r0.6	block01	0
m04	Incogruent	r0.6	block01	0
m04	Incogruent	r0.75	block01	1
m04	Partially Congruent	r0.75	block01	1
m04	Partially Congruent	r0.75	block01	0
m04	Fully Congruent	r0.6	block01	0
m04	Fully Congruent	r0.75	block01	1
m04	Partially Congruent	r0.75	block01	1
m04	Incogruent	r0.6	block01	0
m04	Partially Congruent	r0.75	block01	0
m04	Partially Congruent	r0.6	block01	1
m04	Fully Congruent	r0.75	block01	1
m04	Incogruent	r0.6	block01	0
m04	Partially Congruent	r0.75	block01	0
m04	Fully Congruent	r0.75	block01	1
m04	Fully Congruent	r0.6	block01	1
m04	Partially Congruent	r0.75	block02	0
m04	Fully Congruent	r0.75	block02	1
m04	Incogruent	r0.6	block02	0
m04	Partially Congruent	r0.75	block02	0
m04	Partially Congruent	r0.75	block02	1
m04	Incogruent	r0.6	block02	0
m04	Incogruent	r0.75	block02	1
m04	Fully Congruent	r0.75	block02	1
m04	Fully Congruent	r0.75	block02	1
m04	Partially Congruent	r0.75	block02	0
m04	Fully Congruent	r0.75	block02	0

m04	Partially Congruent	r0.6	block02	1
m04	Incogruent	r0.75	block02	1
m04	Incogruent	r0.6	block02	1
m04	Partially Congruent	r0.6	block02	0
m04	Fully Congruent	r0.75	block02	1
m04	Fully Congruent	r0.75	block02	1
m04	Partially Congruent	r0.75	block02	1
m04	Partially Congruent	r0.75	block02	0
m04	Partially Congruent	r0.6	block02	0
m04	Incogruent	r0.6	block02	0
m04	Partially Congruent	r0.6	block02	0
m04	Incogruent	r0.75	block02	1
m04	Fully Congruent	r0.6	block02	1
m04	Partially Congruent	r0.75	block02	1
m04	Fully Congruent	r0.6	block02	1
m04	Incogruent	r0.6	block02	0
m04	Incogruent	r0.75	block02	0
m04	Fully Congruent	r0.6	block02	1
m04	Partially Congruent	r0.6	block02	1
m04	Fully Congruent	r0.75	block02	0
m04	Fully Congruent	r0.6	block02	0
m04	Fully Congruent	r0.6	block02	1
m04	Partially Congruent	r0.6	block02	1
m04	Fully Congruent	r0.6	block02	1
m04	Fully Congruent	r0.75	block02	0
m04	Partially Congruent	r0.6	block02	1
m04	Fully Congruent	r0.75	block02	1
m04	Fully Congruent	r0.75	block02	0
m04	Partially Congruent	r0.6	block02	0
m04	Incogruent	r0.6	block02	1
m04	Incogruent	r0.6	block02	1
m04	Fully Congruent	r0.75	block02	1
m04	Incogruent	r0.6	block02	0
m04	Fully Congruent	r0.75	block02	1
m04	Partially Congruent	r0.75	block02	1
m04	Partially Congruent	r0.6	block02	1
m04	Partially Congruent	r0.6	block02	1
m04	Fully Congruent	r0.75	block02	0
m04	Fully Congruent	r0.75	block02	0
m04	Fully Congruent	r0.6	block03	1
m04	Incogruent	r0.75	block03	1
m04	Incogruent	r0.6	block03	0
m04	Fully Congruent	r0.75	block03	1
m04	Fully Congruent	r0.6	block03	1
m04	Partially Congruent	r0.6	block03	1

m04	Partially Congruent	r0.6	block03	0
m04	Incogruent	r0.75	block03	0
m04	Partially Congruent	r0.75	block03	1
m04	Partially Congruent	r0.75	block03	1
m04	Partially Congruent	r0.75	block03	0
m04	Partially Congruent	r0.6	block03	0
m04	Partially Congruent	r0.6	block03	0
m04	Partially Congruent	r0.6	block03	1
m04	Fully Congruent	r0.75	block03	1
m04	Incogruent	r0.75	block03	1
m04	Partially Congruent	r0.6	block03	0
m04	Fully Congruent	r0.6	block03	1
m04	Fully Congruent	r0.75	block03	1
m04	Partially Congruent	r0.75	block03	1
m04	Incogruent	r0.75	block03	1
m04	Fully Congruent	r0.75	block03	0
m04	Fully Congruent	r0.6	block03	1
m04	Partially Congruent	r0.6	block03	1
m04	Partially Congruent	r0.6	block03	0
m04	Fully Congruent	r0.75	block03	1
m04	Incogruent	r0.75	block03	1
m04	Incogruent	r0.6	block03	1
m04	Fully Congruent	r0.75	block03	1
m04	Incogruent	r0.75	block03	1
m04	Partially Congruent	r0.6	block03	1
m04	Fully Congruent	r0.75	block03	1
m04	Fully Congruent	r0.75	block03	1
m04	Incogruent	r0.6	block03	0
m04	Partially Congruent	r0.6	block03	1
m04	Partially Congruent	r0.6	block03	0
m04	Incogruent	r0.75	block03	1
m04	Incogruent	r0.6	block03	1
m04	Incogruent	r0.75	block03	0
m04	Incogruent	r0.75	block03	0
m04	Partially Congruent	r0.75	block03	0
m04	Fully Congruent	r0.75	block03	0
m04	Incogruent	r0.6	block03	1
m04	Incogruent	r0.6	block03	0
m04	Fully Congruent	r0.75	block03	1
m04	Fully Congruent	r0.75	block03	1
m04	Fully Congruent	r0.6	block03	0
m04	Partially Congruent	r0.75	block03	0
m04	Fully Congruent	r0.75	block03	1
m04	Incogruent	r0.6	block03	0
m04	Fully Congruent	r0.75	block04	1

m04	Partially Congruent	r0.6	block04	1
m04	Incogruent	r0.75	block04	1
m04	Incogruent	r0.6	block04	0
m04	Incogruent	r0.6	block04	1
m04	Incogruent	r0.75	block04	1
m04	Incogruent	r0.75	block04	0
m04	Fully Congruent	r0.75	block04	0
m04	Incogruent	r0.75	block04	0
m04	Incogruent	r0.75	block04	0
m04	Partially Congruent	r0.6	block04	1
m04	Incogruent	r0.75	block04	1
m04	Fully Congruent	r0.75	block04	1
m04	Incogruent	r0.75	block04	0
m04	Incogruent	r0.6	block04	1
m04	Incogruent	r0.6	block04	0
m04	Partially Congruent	r0.6	block04	0
m04	Fully Congruent	r0.6	block04	0
m04	Incogruent	r0.6	block04	1
m04	Partially Congruent	r0.75	block04	1
m04	Fully Congruent	r0.6	block04	1
m04	Incogruent	r0.75	block04	0
m04	Partially Congruent	r0.6	block04	1
m04	Incogruent	r0.6	block04	0
m04	Incogruent	r0.75	block04	1
m04	Partially Congruent	r0.75	block04	0
m04	Fully Congruent	r0.75	block04	1
m04	Partially Congruent	r0.75	block04	1
m04	Incogruent	r0.6	block04	0
m04	Fully Congruent	r0.6	block04	1
m04	Partially Congruent	r0.6	block04	1
m04	Fully Congruent	r0.75	block04	1
m04	Fully Congruent	r0.75	block04	1
m04	Fully Congruent	r0.75	block04	1
m04	Incogruent	r0.75	block04	1
m04	Incogruent	r0.6	block04	1
m04	Incogruent	r0.75	block04	1
m04	Fully Congruent	r0.75	block04	0
m04	Fully Congruent	r0.6	block04	1
m04	Incogruent	r0.75	block04	0
m04	Partially Congruent	r0.75	block04	0
m04	Fully Congruent	r0.75	block04	1
m04	Partially Congruent	r0.75	block04	0
m04	Fully Congruent	r0.6	block04	0
m04	Incogruent	r0.75	block04	1
m04	Incogruent	r0.75	block04	0

m04	Fully Congruent	r0.75	block04	0
m04	Fully Congruent	r0.75	block04	1
m04	Fully Congruent	r0.75	block04	1
m04	Incogruent	r0.75	block04	0
m04	Partially Congruent	r0.6	block05	0
m04	Fully Congruent	r0.6	block05	1
m04	Partially Congruent	r0.6	block05	1
m04	Incogruent	r0.6	block05	0
m04	Partially Congruent	r0.6	block05	0
m04	Incogruent	r0.75	block05	1
m04	Fully Congruent	r0.75	block05	0
m04	Fully Congruent	r0.6	block05	1
m04	Incogruent	r0.75	block05	1
m04	Fully Congruent	r0.75	block05	1
m04	Incogruent	r0.6	block05	1
m04	Incogruent	r0.75	block05	0
m04	Partially Congruent	r0.6	block05	0
m04	Incogruent	r0.75	block05	1
m04	Fully Congruent	r0.75	block05	1
m04	Fully Congruent	r0.75	block05	1
m04	Fully Congruent	r0.6	block05	1
m04	Incogruent	r0.6	block05	0
m04	Partially Congruent	r0.75	block05	0
m04	Partially Congruent	r0.75	block05	1
m04	Partially Congruent	r0.75	block05	0
m04	Partially Congruent	r0.6	block05	0
m04	Incogruent	r0.75	block05	0
m04	Incogruent	r0.6	block05	1
m04	Fully Congruent	r0.6	block05	0
m04	Fully Congruent	r0.6	block05	1
m04	Fully Congruent	r0.6	block05	1
m04	Incogruent	r0.6	block05	0
m04	Partially Congruent	r0.75	block05	1
m04	Fully Congruent	r0.75	block05	1
m04	Incogruent	r0.75	block05	1
m04	Fully Congruent	r0.6	block05	1
m04	Fully Congruent	r0.6	block05	1
m04	Incogruent	r0.6	block05	1
m04	Partially Congruent	r0.75	block05	0
m04	Fully Congruent	r0.6	block05	0
m04	Incogruent	r0.75	block05	0
m04	Fully Congruent	r0.75	block05	1
m04	Fully Congruent	r0.6	block05	1
m04	Incogruent	r0.6	block05	1
m04	Partially Congruent	r0.6	block05	0

m04	Fully Congruent	r0.75	block05	0
m04	Fully Congruent	r0.75	block05	0
m04	Fully Congruent	r0.6	block05	1
m04	Fully Congruent	r0.75	block05	1
m04	Fully Congruent	r0.6	block05	1
m04	Partially Congruent	r0.75	block05	1
m04	Incogruent	r0.6	block05	0
m04	Fully Congruent	r0.75	block05	1
m04	Fully Congruent	r0.75	block05	1
m04	Incogruent	r0.6	block06	1
m04	Fully Congruent	r0.6	block06	0
m04	Partially Congruent	r0.75	block06	1
m04	Fully Congruent	r0.75	block06	1
m04	Incogruent	r0.75	block06	1
m04	Incogruent	r0.75	block06	0
m04	Incogruent	r0.75	block06	0
m04	Partially Congruent	r0.6	block06	1
m04	Fully Congruent	r0.75	block06	0
m04	Fully Congruent	r0.75	block06	1
m04	Incogruent	r0.6	block06	1
m04	Fully Congruent	r0.6	block06	1
m04	Incogruent	r0.6	block06	0
m04	Fully Congruent	r0.6	block06	1
m04	Incogruent	r0.75	block06	1
m04	Fully Congruent	r0.75	block06	0
m04	Incogruent	r0.75	block06	1
m04	Fully Congruent	r0.75	block06	0
m04	Partially Congruent	r0.75	block06	1
m04	Fully Congruent	r0.6	block06	1
m04	Incogruent	r0.6	block06	0
m04	Incogruent	r0.75	block06	0
m04	Partially Congruent	r0.6	block06	0
m04	Fully Congruent	r0.75	block06	0
m04	Partially Congruent	r0.6	block06	1
m04	Partially Congruent	r0.6	block06	1
m04	Partially Congruent	r0.6	block06	1
m04	Partially Congruent	r0.75	block06	1
m04	Partially Congruent	r0.6	block06	1
m04	Fully Congruent	r0.75	block06	1
m04	Incogruent	r0.75	block06	0
m04	Fully Congruent	r0.75	block06	1
m04	Incogruent	r0.6	block06	0
m04	Incogruent	r0.75	block06	1
m04	Partially Congruent	r0.75	block06	0
m04	Fully Congruent	r0.6	block06	1

m04	Partially Congruent	r0.75	block06	0
m04	Fully Congruent	r0.6	block06	1
m04	Partially Congruent	r0.75	block06	0
m04	Partially Congruent	r0.75	block06	0
m04	Incogruent	r0.75	block06	1
m04	Partially Congruent	r0.6	block06	1
m04	Incogruent	r0.75	block06	0
m04	Fully Congruent	r0.75	block06	0
m04	Fully Congruent	r0.75	block06	1
m04	Fully Congruent	r0.6	block06	1
m04	Incogruent	r0.6	block06	1
m04	Partially Congruent	r0.75	block06	0
m04	Fully Congruent	r0.75	block06	0
m04	Incogruent	r0.75	block06	1
m04	Incogruent	r0.75	block07	0
m04	Fully Congruent	r0.75	block07	1
m04	Partially Congruent	r0.6	block07	0
m04	Partially Congruent	r0.75	block07	0
m04	Partially Congruent	r0.75	block07	1
m04	Fully Congruent	r0.6	block07	1
m04	Incogruent	r0.75	block07	0
m04	Incogruent	r0.75	block07	1
m04	Partially Congruent	r0.75	block07	1
m04	Fully Congruent	r0.75	block07	0
m04	Incogruent	r0.75	block07	1
m04	Partially Congruent	r0.75	block07	1
m04	Incogruent	r0.6	block07	1
m04	Fully Congruent	r0.75	block07	1
m04	Fully Congruent	r0.75	block07	1
m04	Partially Congruent	r0.75	block07	0
m04	Incogruent	r0.75	block07	1
m04	Fully Congruent	r0.6	block07	1
m04	Fully Congruent	r0.6	block07	1
m04	Partially Congruent	r0.75	block07	0
m04	Incogruent	r0.6	block07	0
m04	Fully Congruent	r0.6	block07	1
m04	Partially Congruent	r0.6	block07	1
m04	Partially Congruent	r0.75	block07	1
m04	Incogruent	r0.6	block07	0
m04	Fully Congruent	r0.75	block07	0
m04	Partially Congruent	r0.75	block07	0
m04	Incogruent	r0.75	block07	0
m04	Partially Congruent	r0.75	block07	1
m04	Partially Congruent	r0.75	block07	1
m04	Incogruent	r0.6	block07	1

m04	Fully Congruent	r0.6	block07	1
m04	Partially Congruent	r0.75	block07	1
m04	Fully Congruent	r0.75	block07	1
m04	Fully Congruent	r0.75	block07	0
m04	Partially Congruent	r0.75	block07	1
m04	Incogruent	r0.6	block07	0
m04	Incogruent	r0.6	block07	0
m04	Incogruent	r0.75	block07	0
m04	Incogruent	r0.75	block07	0
m04	Fully Congruent	r0.6	block07	1
m04	Partially Congruent	r0.75	block07	1
m04	Partially Congruent	r0.6	block07	0
m04	Incogruent	r0.75	block07	1
m04	Incogruent	r0.6	block07	1
m04	Partially Congruent	r0.6	block07	1
m04	Fully Congruent	r0.6	block07	1
m04	Incogruent	r0.75	block07	1
m04	Incogruent	r0.6	block07	0
m04	Partially Congruent	r0.75	block07	1
m04	Partially Congruent	r0.75	block08	0
m04	Fully Congruent	r0.75	block08	1
m04	Fully Congruent	r0.75	block08	0
m04	Incogruent	r0.75	block08	0
m04	Partially Congruent	r0.6	block08	0
m04	Fully Congruent	r0.6	block08	0
m04	Incogruent	r0.75	block08	0
m04	Partially Congruent	r0.6	block08	1
m04	Incogruent	r0.6	block08	1
m04	Incogruent	r0.6	block08	0
m04	Incogruent	r0.75	block08	1
m04	Partially Congruent	r0.6	block08	1
m04	Incogruent	r0.6	block08	1
m04	Fully Congruent	r0.75	block08	0
m04	Incogruent	r0.6	block08	1
m04	Partially Congruent	r0.6	block08	1
m04	Fully Congruent	r0.75	block08	1
m04	Partially Congruent	r0.75	block08	1
m04	Partially Congruent	r0.75	block08	0
m04	Partially Congruent	r0.75	block08	1
m04	Incogruent	r0.75	block08	1
m04	Fully Congruent	r0.6	block08	0
m04	Fully Congruent	r0.75	block08	1
m04	Incogruent	r0.6	block08	0
m04	Partially Congruent	r0.75	block08	0
m04	Fully Congruent	r0.75	block08	1

m04	Fully Congruent	r0.75	block08	1
m04	Incogruent	r0.6	block08	1
m04	Fully Congruent	r0.75	block08	1
m04	Fully Congruent	r0.6	block08	0
m04	Incogruent	r0.6	block08	1
m04	Fully Congruent	r0.75	block08	0
m04	Fully Congruent	r0.75	block08	1
m04	Incogruent	r0.75	block08	0
m04	Partially Congruent	r0.6	block08	1
m04	Incogruent	r0.75	block08	1
m04	Partially Congruent	r0.75	block08	0
m04	Fully Congruent	r0.75	block08	1
m04	Fully Congruent	r0.6	block08	1
m04	Fully Congruent	r0.75	block08	0
m04	Incogruent	r0.75	block08	1
m04	Partially Congruent	r0.6	block08	0
m04	Partially Congruent	r0.75	block08	1
m04	Incogruent	r0.75	block08	1
m04	Partially Congruent	r0.6	block08	1
m04	Fully Congruent	r0.75	block08	1
m04	Incogruent	r0.75	block08	0
m04	Partially Congruent	r0.75	block08	1
m04	Incogruent	r0.6	block08	1
m04	Partially Congruent	r0.6	block08	0
m04	Partially Congruent	r0.6	block09	1
m04	Fully Congruent	r0.75	block09	0
m04	Incogruent	r0.75	block09	1
m04	Partially Congruent	r0.6	block09	1
m04	Fully Congruent	r0.75	block09	0
m04	Fully Congruent	r0.75	block09	1
m04	Fully Congruent	r0.75	block09	1
m04	Partially Congruent	r0.6	block09	1
m04	Incogruent	r0.75	block09	0
m04	Incogruent	r0.75	block09	0
m04	Incogruent	r0.75	block09	1
m04	Fully Congruent	r0.75	block09	0
m04	Incogruent	r0.6	block09	0
m04	Fully Congruent	r0.75	block09	1
m04	Incogruent	r0.6	block09	0
m04	Partially Congruent	r0.6	block09	1
m04	Fully Congruent	r0.75	block09	1
m04	Incogruent	r0.75	block09	0
m04	Incogruent	r0.75	block09	0
m04	Fully Congruent	r0.6	block09	1
m04	Partially Congruent	r0.6	block09	1

m04	Partially Congruent	r0.75	block09	0
m04	Partially Congruent	r0.6	block09	0
m04	Incogruent	r0.75	block09	0
m04	Fully Congruent	r0.75	block09	1
m04	Fully Congruent	r0.6	block09	0
m04	Incogruent	r0.75	block09	0
m04	Partially Congruent	r0.6	block09	1
m04	Fully Congruent	r0.75	block09	0
m04	Partially Congruent	r0.75	block09	0
m04	Partially Congruent	r0.75	block09	0
m04	Partially Congruent	r0.6	block09	0
m04	Partially Congruent	r0.6	block09	1
m04	Partially Congruent	r0.6	block09	0
m04	Partially Congruent	r0.6	block09	0
m04	Partially Congruent	r0.6	block09	0
m04	Incogruent	r0.6	block09	1
m04	Partially Congruent	r0.6	block09	0
m04	Fully Congruent	r0.75	block09	1
m04	Incogruent	r0.6	block09	1
m04	Partially Congruent	r0.75	block09	1
m04	Incogruent	r0.6	block09	1
m04	Partially Congruent	r0.6	block09	0
m04	Incogruent	r0.6	block09	1
m04	Fully Congruent	r0.75	block09	1
m04	Partially Congruent	r0.75	block09	0
m04	Partially Congruent	r0.75	block09	1
m04	Fully Congruent	r0.75	block09	1
m04	Fully Congruent	r0.75	block09	0
m04	Incogruent	r0.75	block09	1
m04	Fully Congruent	r0.75	block10	1
m04	Incogruent	r0.6	block10	1
m04	Fully Congruent	r0.75	block10	0
m04	Partially Congruent	r0.6	block10	1
m04	Fully Congruent	r0.75	block10	1
m04	Partially Congruent	r0.6	block10	0
m04	Fully Congruent	r0.75	block10	1
m04	Partially Congruent	r0.75	block10	1
m04	Incogruent	r0.6	block10	0
m04	Partially Congruent	r0.75	block10	0
m04	Partially Congruent	r0.6	block10	1
m04	Partially Congruent	r0.6	block10	0
m04	Partially Congruent	r0.6	block10	1
m04	Incogruent	r0.6	block10	1
m04	Partially Congruent	r0.75	block10	1
m04	Incogruent	r0.6	block10	1

m04	Incogruent	r0.75	block10	0
m04	Partially Congruent	r0.75	block10	0
m04	Fully Congruent	r0.6	block10	0
m04	Partially Congruent	r0.6	block10	1
m04	Fully Congruent	r0.75	block10	1
m04	Incogruent	r0.75	block10	0
m04	Partially Congruent	r0.75	block10	0
m04	Partially Congruent	r0.6	block10	1
m04	Fully Congruent	r0.75	block10	0
m04	Partially Congruent	r0.75	block10	0
m04	Fully Congruent	r0.75	block10	1
m04	Fully Congruent	r0.6	block10	1
m04	Partially Congruent	r0.6	block10	0
m04	Incogruent	r0.6	block10	0
m04	Incogruent	r0.6	block10	1
m04	Partially Congruent	r0.6	block10	0
m04	Incogruent	r0.6	block10	0
m04	Incogruent	r0.6	block10	1
m04	Fully Congruent	r0.75	block10	0
m04	Fully Congruent	r0.6	block10	1
m04	Incogruent	r0.6	block10	1
m04	Incogruent	r0.75	block10	0
m04	Fully Congruent	r0.6	block10	1
m04	Partially Congruent	r0.75	block10	1
m04	Fully Congruent	r0.75	block10	1
m04	Partially Congruent	r0.75	block10	1
m04	Fully Congruent	r0.6	block10	0
m04	Incogruent	r0.75	block10	1
m04	Fully Congruent	r0.6	block10	0
m04	Incogruent	r0.6	block10	0
m04	Incogruent	r0.6	block10	1
m04	Incogruent	r0.75	block10	0
m04	Fully Congruent	r0.75	block10	1
m04	Partially Congruent	r0.75	block10	0
m01	Incogruent	r0.75	block01	0
m01	Incogruent	r0.75	block01	1
m01	Fully Congruent	r0.75	block01	1
m01	Incogruent	r0.75	block01	0
m01	Incogruent	r0.75	block01	0
m01	Partially Congruent	r0.6	block01	0
m01	Partially Congruent	r0.6	block01	0
m01	Partially Congruent	r0.6	block01	0
m01	Fully Congruent	r0.75	block01	0
m01	Fully Congruent	r0.75	block01	1
m01	Partially Congruent	r0.6	block01	1

m01	Incogruent	r0.75	block01	1
m01	Incogruent	r0.6	block01	0
m01	Partially Congruent	r0.75	block01	0
m01	Incogruent	r0.6	block01	0
m01	Fully Congruent	r0.6	block01	1
m01	Fully Congruent	r0.75	block01	0
m01	Fully Congruent	r0.6	block01	0
m01	Fully Congruent	r0.75	block01	1
m01	Fully Congruent	r0.75	block01	0
m01	Partially Congruent	r0.6	block01	0
m01	Incogruent	r0.75	block01	1
m01	Fully Congruent	r0.6	block01	0
m01	Partially Congruent	r0.6	block01	1
m01	Fully Congruent	r0.75	block01	1
m01	Partially Congruent	r0.75	block01	0
m01	Partially Congruent	r0.6	block01	0
m01	Incogruent	r0.6	block01	0
m01	Incogruent	r0.6	block01	1
m01	Fully Congruent	r0.75	block01	1
m01	Fully Congruent	r0.6	block01	0
m01	Fully Congruent	r0.75	block01	1
m01	Fully Congruent	r0.75	block01	0
m01	Partially Congruent	r0.75	block01	0
m01	Partially Congruent	r0.6	block01	0
m01	Incogruent	r0.6	block01	1
m01	Incogruent	r0.75	block01	0
m01	Incogruent	r0.75	block01	0
m01	Incogruent	r0.6	block01	0
m01	Incogruent	r0.6	block01	1
m01	Incogruent	r0.75	block01	1
m01	Fully Congruent	r0.75	block01	1
m01	Incogruent	r0.75	block01	0
m01	Fully Congruent	r0.75	block01	0
m01	Incogruent	r0.6	block01	1
m01	Fully Congruent	r0.75	block01	1
m01	Incogruent	r0.6	block01	0
m01	Fully Congruent	r0.6	block01	0
m01	Incogruent	r0.6	block01	1
m01	Partially Congruent	r0.75	block01	1
m01	Partially Congruent	r0.75	block02	1
m01	Fully Congruent	r0.75	block02	0
m01	Partially Congruent	r0.75	block02	0
m01	Incogruent	r0.75	block02	0
m01	Partially Congruent	r0.75	block02	1
m01	Incogruent	r0.6	block02	1

m01	Partially Congruent	r0.6	block02	1
m01	Incogruent	r0.6	block02	1
m01	Partially Congruent	r0.75	block02	1
m01	Incogruent	r0.6	block02	1
m01	Fully Congruent	r0.6	block02	1
m01	Partially Congruent	r0.6	block02	0
m01	Incogruent	r0.6	block02	1
m01	Incogruent	r0.6	block02	1
m01	Fully Congruent	r0.6	block02	0
m01	Fully Congruent	r0.75	block02	1
m01	Fully Congruent	r0.75	block02	1
m01	Partially Congruent	r0.6	block02	0
m01	Partially Congruent	r0.6	block02	1
m01	Fully Congruent	r0.75	block02	1
m01	Incogruent	r0.75	block02	1
m01	Partially Congruent	r0.75	block02	1
m01	Partially Congruent	r0.6	block02	0
m01	Fully Congruent	r0.75	block02	0
m01	Partially Congruent	r0.6	block02	1
m01	Incogruent	r0.6	block02	0
m01	Partially Congruent	r0.75	block02	0
m01	Fully Congruent	r0.75	block02	1
m01	Incogruent	r0.6	block02	0
m01	Partially Congruent	r0.6	block02	0
m01	Partially Congruent	r0.75	block02	1
m01	Partially Congruent	r0.6	block02	1
m01	Fully Congruent	r0.75	block02	0
m01	Partially Congruent	r0.6	block02	1
m01	Partially Congruent	r0.6	block02	1
m01	Partially Congruent	r0.75	block02	1
m01	Incogruent	r0.75	block02	0
m01	Partially Congruent	r0.6	block02	1
m01	Partially Congruent	r0.6	block02	1
m01	Fully Congruent	r0.6	block02	1
m01	Fully Congruent	r0.75	block02	1
m01	Partially Congruent	r0.6	block02	1
m01	Partially Congruent	r0.75	block02	0
m01	Partially Congruent	r0.6	block02	1
m01	Incogruent	r0.6	block02	0
m01	Fully Congruent	r0.75	block02	1
m01	Incogruent	r0.75	block02	0
m01	Fully Congruent	r0.75	block02	1
m01	Incogruent	r0.6	block02	1
m01	Partially Congruent	r0.6	block02	0
m01	Partially Congruent	r0.6	block03	0

m01	Incogruent	r0.6	block03	0
m01	Partially Congruent	r0.75	block03	1
m01	Incogruent	r0.75	block03	1
m01	Fully Congruent	r0.75	block03	1
m01	Incogruent	r0.6	block03	1
m01	Partially Congruent	r0.6	block03	0
m01	Incogruent	r0.75	block03	1
m01	Fully Congruent	r0.75	block03	1
m01	Fully Congruent	r0.6	block03	0
m01	Partially Congruent	r0.6	block03	1
m01	Partially Congruent	r0.75	block03	1
m01	Partially Congruent	r0.6	block03	1
m01	Fully Congruent	r0.75	block03	1
m01	Fully Congruent	r0.6	block03	0
m01	Fully Congruent	r0.75	block03	1
m01	Partially Congruent	r0.6	block03	0
m01	Fully Congruent	r0.75	block03	0
m01	Incogruent	r0.75	block03	0
m01	Incogruent	r0.75	block03	1
m01	Partially Congruent	r0.6	block03	1
m01	Fully Congruent	r0.75	block03	1
m01	Fully Congruent	r0.75	block03	1
m01	Partially Congruent	r0.6	block03	0
m01	Partially Congruent	r0.75	block03	0
m01	Incogruent	r0.6	block03	0
m01	Partially Congruent	r0.75	block03	1
m01	Incogruent	r0.6	block03	1
m01	Fully Congruent	r0.75	block03	1
m01	Partially Congruent	r0.6	block03	1
m01	Fully Congruent	r0.6	block03	0
m01	Fully Congruent	r0.75	block03	1
m01	Partially Congruent	r0.6	block03	0
m01	Incogruent	r0.6	block03	0
m01	Fully Congruent	r0.75	block03	1
m01	Fully Congruent	r0.75	block03	0
m01	Fully Congruent	r0.75	block03	1
m01	Incogruent	r0.6	block03	1
m01	Fully Congruent	r0.75	block03	0
m01	Fully Congruent	r0.6	block03	1
m01	Fully Congruent	r0.75	block03	1
m01	Incogruent	r0.75	block03	1
m01	Incogruent	r0.6	block03	0
m01	Incogruent	r0.75	block03	0
m01	Incogruent	r0.75	block03	0
m01	Incogruent	r0.6	block03	0

m01	Partially Congruent	r0.6	block03	1
m01	Fully Congruent	r0.75	block03	1
m01	Partially Congruent	r0.6	block03	1
m01	Incogruent	r0.6	block03	0
m01	Fully Congruent	r0.6	block04	0
m01	Partially Congruent	r0.6	block04	0
m01	Fully Congruent	r0.75	block04	1
m01	Incogruent	r0.6	block04	1
m01	Fully Congruent	r0.75	block04	0
m01	Fully Congruent	r0.75	block04	1
m01	Fully Congruent	r0.75	block04	1
m01	Incogruent	r0.6	block04	0
m01	Partially Congruent	r0.75	block04	1
m01	Incogruent	r0.75	block04	1
m01	Partially Congruent	r0.6	block04	1
m01	Fully Congruent	r0.75	block04	1
m01	Fully Congruent	r0.6	block04	1
m01	Partially Congruent	r0.75	block04	0
m01	Incogruent	r0.6	block04	1
m01	Partially Congruent	r0.75	block04	1
m01	Partially Congruent	r0.6	block04	0
m01	Partially Congruent	r0.75	block04	0
m01	Incogruent	r0.75	block04	0
m01	Incogruent	r0.6	block04	0
m01	Fully Congruent	r0.75	block04	1
m01	Partially Congruent	r0.6	block04	1
m01	Fully Congruent	r0.6	block04	1
m01	Incogruent	r0.75	block04	1
m01	Partially Congruent	r0.75	block04	0
m01	Incogruent	r0.6	block04	0
m01	Fully Congruent	r0.75	block04	1
m01	Partially Congruent	r0.6	block04	1
m01	Partially Congruent	r0.75	block04	0
m01	Incogruent	r0.75	block04	1
m01	Incogruent	r0.6	block04	1
m01	Incogruent	r0.75	block04	1
m01	Partially Congruent	r0.75	block04	0
m01	Fully Congruent	r0.6	block04	1
m01	Partially Congruent	r0.6	block04	0
m01	Incogruent	r0.6	block04	0
m01	Fully Congruent	r0.75	block04	1
m01	Partially Congruent	r0.75	block04	0
m01	Partially Congruent	r0.75	block04	0
m01	Fully Congruent	r0.6	block04	1
m01	Incogruent	r0.75	block04	0

m01	Incogruent	r0.75	block04	0
m01	Partially Congruent	r0.75	block04	0
m01	Fully Congruent	r0.75	block04	0
m01	Fully Congruent	r0.75	block04	1
m01	Fully Congruent	r0.75	block04	1
m01	Partially Congruent	r0.75	block04	0
m01	Partially Congruent	r0.6	block04	1
m01	Partially Congruent	r0.75	block04	1
m01	Partially Congruent	r0.75	block04	0
m01	Fully Congruent	r0.75	block05	1
m01	Incogruent	r0.75	block05	0
m01	Partially Congruent	r0.6	block05	1
m01	Fully Congruent	r0.75	block05	1
m01	Partially Congruent	r0.6	block05	1
m01	Incogruent	r0.6	block05	0
m01	Partially Congruent	r0.6	block05	1
m01	Partially Congruent	r0.6	block05	0
m01	Partially Congruent	r0.75	block05	1
m01	Partially Congruent	r0.75	block05	0
m01	Partially Congruent	r0.75	block05	1
m01	Fully Congruent	r0.75	block05	0
m01	Partially Congruent	r0.6	block05	0
m01	Incogruent	r0.6	block05	0
m01	Partially Congruent	r0.75	block05	1
m01	Fully Congruent	r0.75	block05	0
m01	Fully Congruent	r0.75	block05	1
m01	Partially Congruent	r0.6	block05	0
m01	Fully Congruent	r0.6	block05	1
m01	Fully Congruent	r0.6	block05	0
m01	Incogruent	r0.75	block05	1
m01	Partially Congruent	r0.75	block05	0
m01	Partially Congruent	r0.6	block05	0
m01	Fully Congruent	r0.6	block05	0
m01	Incogruent	r0.75	block05	1
m01	Fully Congruent	r0.75	block05	1
m01	Fully Congruent	r0.75	block05	1
m01	Fully Congruent	r0.6	block05	1
m01	Fully Congruent	r0.75	block05	1
m01	Partially Congruent	r0.75	block05	0
m01	Incogruent	r0.6	block05	1
m01	Partially Congruent	r0.75	block05	1
m01	Incogruent	r0.6	block05	1
m01	Incogruent	r0.75	block05	0
m01	Partially Congruent	r0.6	block05	1
m01	Incogruent	r0.75	block05	1

m01	Incogruent	r0.75	block05	0
m01	Partially Congruent	r0.6	block05	1
m01	Fully Congruent	r0.75	block05	1
m01	Incogruent	r0.75	block05	0
m01	Incogruent	r0.6	block05	0
m01	Fully Congruent	r0.75	block05	0
m01	Incogruent	r0.75	block05	0
m01	Fully Congruent	r0.75	block05	0
m01	Fully Congruent	r0.75	block05	1
m01	Fully Congruent	r0.75	block05	0
m01	Fully Congruent	r0.6	block05	1
m01	Partially Congruent	r0.6	block05	1
m01	Fully Congruent	r0.75	block05	0
m01	Incogruent	r0.6	block05	0
m01	Partially Congruent	r0.75	block06	0
m01	Partially Congruent	r0.6	block06	1
m01	Fully Congruent	r0.75	block06	0
m01	Partially Congruent	r0.6	block06	0
m01	Incogruent	r0.75	block06	1
m01	Incogruent	r0.75	block06	1
m01	Fully Congruent	r0.75	block06	1
m01	Fully Congruent	r0.75	block06	0
m01	Fully Congruent	r0.75	block06	1
m01	Partially Congruent	r0.75	block06	0
m01	Incogruent	r0.6	block06	1
m01	Incogruent	r0.75	block06	1
m01	Incogruent	r0.75	block06	1
m01	Incogruent	r0.75	block06	1
m01	Incogruent	r0.6	block06	0
m01	Incogruent	r0.75	block06	0
m01	Incogruent	r0.75	block06	0
m01	Incogruent	r0.6	block06	0
m01	Incogruent	r0.6	block06	1
m01	Partially Congruent	r0.6	block06	0
m01	Incogruent	r0.6	block06	0
m01	Fully Congruent	r0.75	block06	1
m01	Partially Congruent	r0.6	block06	0
m01	Incogruent	r0.6	block06	0
m01	Incogruent	r0.75	block06	1
m01	Fully Congruent	r0.6	block06	1
m01	Incogruent	r0.6	block06	1
m01	Fully Congruent	r0.75	block06	0
m01	Incogruent	r0.75	block06	0
m01	Partially Congruent	r0.6	block06	1
m01	Incogruent	r0.75	block06	1

m01	Incogruent	r0.75	block06	1
m01	Fully Congruent	r0.75	block06	1
m01	Fully Congruent	r0.6	block06	0
m01	Partially Congruent	r0.6	block06	1
m01	Partially Congruent	r0.75	block06	1
m01	Partially Congruent	r0.6	block06	1
m01	Partially Congruent	r0.6	block06	0
m01	Fully Congruent	r0.75	block06	1
m01	Incogruent	r0.75	block06	1
m01	Incogruent	r0.6	block06	1
m01	Incogruent	r0.6	block06	1
m01	Partially Congruent	r0.6	block06	0
m01	Fully Congruent	r0.75	block06	1
m01	Fully Congruent	r0.6	block06	1
m01	Fully Congruent	r0.75	block06	0
m01	Partially Congruent	r0.6	block06	1
m01	Fully Congruent	r0.75	block06	1
m01	Partially Congruent	r0.6	block06	1
m01	Partially Congruent	r0.6	block06	0
m01	Incogruent	r0.75	block07	0
m01	Incogruent	r0.6	block07	1
m01	Partially Congruent	r0.6	block07	0
m01	Partially Congruent	r0.6	block07	1
m01	Incogruent	r0.75	block07	1
m01	Incogruent	r0.6	block07	0
m01	Partially Congruent	r0.6	block07	0
m01	Fully Congruent	r0.75	block07	1
m01	Partially Congruent	r0.6	block07	1
m01	Partially Congruent	r0.6	block07	0
m01	Fully Congruent	r0.75	block07	0
m01	Fully Congruent	r0.75	block07	1
m01	Incogruent	r0.6	block07	1
m01	Partially Congruent	r0.6	block07	1
m01	Fully Congruent	r0.75	block07	0
m01	Fully Congruent	r0.6	block07	0
m01	Incogruent	r0.6	block07	0
m01	Incogruent	r0.6	block07	1
m01	Incogruent	r0.6	block07	1
m01	Fully Congruent	r0.6	block07	0
m01	Fully Congruent	r0.6	block07	0
m01	Fully Congruent	r0.75	block07	1
m01	Incogruent	r0.6	block07	0
m01	Fully Congruent	r0.75	block07	1
m01	Partially Congruent	r0.75	block07	1
m01	Fully Congruent	r0.75	block07	1

m01	Fully Congruent	r0.75	block07	0
m01	Fully Congruent	r0.6	block07	0
m01	Fully Congruent	r0.6	block07	1
m01	Fully Congruent	r0.75	block07	1
m01	Incogruent	r0.6	block07	1
m01	Fully Congruent	r0.75	block07	1
m01	Incogruent	r0.75	block07	1
m01	Fully Congruent	r0.75	block07	1
m01	Partially Congruent	r0.75	block07	1
m01	Fully Congruent	r0.75	block07	0
m01	Partially Congruent	r0.75	block07	0
m01	Fully Congruent	r0.75	block07	0
m01	Incogruent	r0.75	block07	1
m01	Incogruent	r0.6	block07	0
m01	Incogruent	r0.6	block07	0
m01	Fully Congruent	r0.6	block07	1
m01	Fully Congruent	r0.75	block07	1
m01	Partially Congruent	r0.6	block07	0
m01	Partially Congruent	r0.6	block07	0
m01	Partially Congruent	r0.6	block07	0
m01	Incogruent	r0.75	block07	0
m01	Partially Congruent	r0.6	block07	1
m01	Partially Congruent	r0.6	block07	1
m01	Partially Congruent	r0.6	block07	0
m01	Partially Congruent	r0.6	block08	0
m01	Fully Congruent	r0.75	block08	0
m01	Fully Congruent	r0.75	block08	0
m01	Fully Congruent	r0.75	block08	1
m01	Partially Congruent	r0.6	block08	0
m01	Fully Congruent	r0.75	block08	1
m01	Fully Congruent	r0.75	block08	1
m01	Fully Congruent	r0.75	block08	1
m01	Partially Congruent	r0.75	block08	0
m01	Fully Congruent	r0.6	block08	1
m01	Partially Congruent	r0.75	block08	1
m01	Partially Congruent	r0.6	block08	1
m01	Incogruent	r0.75	block08	1
m01	Partially Congruent	r0.75	block08	1
m01	Incogruent	r0.75	block08	0
m01	Partially Congruent	r0.75	block08	0
m01	Fully Congruent	r0.6	block08	1
m01	Incogruent	r0.6	block08	0
m01	Fully Congruent	r0.75	block08	1
m01	Partially Congruent	r0.6	block08	1
m01	Partially Congruent	r0.75	block08	0

m01	Partially Congruent	r0.6	block08	0
m01	Incogruent	r0.6	block08	1
m01	Fully Congruent	r0.75	block08	1
m01	Partially Congruent	r0.75	block08	1
m01	Fully Congruent	r0.75	block08	1
m01	Partially Congruent	r0.6	block08	0
m01	Fully Congruent	r0.75	block08	0
m01	Incogruent	r0.6	block08	0
m01	Partially Congruent	r0.6	block08	0
m01	Fully Congruent	r0.75	block08	1
m01	Fully Congruent	r0.75	block08	1
m01	Partially Congruent	r0.75	block08	0
m01	Incogruent	r0.6	block08	0
m01	Incogruent	r0.6	block08	1
m01	Fully Congruent	r0.75	block08	0
m01	Partially Congruent	r0.6	block08	0
m01	Incogruent	r0.6	block08	0
m01	Incogruent	r0.6	block08	1
m01	Incogruent	r0.6	block08	1
m01	Incogruent	r0.75	block08	1
m01	Partially Congruent	r0.6	block08	1
m01	Incogruent	r0.6	block08	0
m01	Incogruent	r0.6	block08	0
m01	Fully Congruent	r0.6	block08	0
m01	Incogruent	r0.75	block08	0
m01	Partially Congruent	r0.75	block08	0
m01	Partially Congruent	r0.75	block08	0
m01	Partially Congruent	r0.6	block08	0
m01	Incogruent	r0.6	block08	1
m01	Partially Congruent	r0.6	block09	1
m01	Fully Congruent	r0.75	block09	0
m01	Incogruent	r0.6	block09	1
m01	Partially Congruent	r0.75	block09	0
m01	Partially Congruent	r0.6	block09	1
m01	Partially Congruent	r0.75	block09	0
m01	Partially Congruent	r0.6	block09	1
m01	Fully Congruent	r0.75	block09	0
m01	Incogruent	r0.75	block09	1
m01	Fully Congruent	r0.75	block09	0
m01	Partially Congruent	r0.6	block09	1
m01	Partially Congruent	r0.6	block09	1
m01	Partially Congruent	r0.75	block09	0
m01	Fully Congruent	r0.75	block09	0
m01	Partially Congruent	r0.6	block09	0
m01	Incogruent	r0.6	block09	1

m01	Incogruent	r0.6	block09	1
m01	Partially Congruent	r0.75	block09	0
m01	Fully Congruent	r0.75	block09	1
m01	Incogruent	r0.6	block09	0
m01	Partially Congruent	r0.6	block09	0
m01	Incogruent	r0.6	block09	0
m01	Fully Congruent	r0.75	block09	1
m01	Partially Congruent	r0.6	block09	0
m01	Fully Congruent	r0.75	block09	1
m01	Incogruent	r0.75	block09	1
m01	Partially Congruent	r0.75	block09	1
m01	Fully Congruent	r0.75	block09	0
m01	Fully Congruent	r0.6	block09	1
m01	Partially Congruent	r0.6	block09	0
m01	Fully Congruent	r0.6	block09	0
m01	Incogruent	r0.6	block09	1
m01	Incogruent	r0.6	block09	0
m01	Partially Congruent	r0.75	block09	1
m01	Incogruent	r0.6	block09	1
m01	Partially Congruent	r0.6	block09	0
m01	Partially Congruent	r0.75	block09	1
m01	Fully Congruent	r0.75	block09	0
m01	Incogruent	r0.6	block09	0
m01	Partially Congruent	r0.6	block09	1
m01	Fully Congruent	r0.75	block09	1
m01	Partially Congruent	r0.6	block09	0
m01	Partially Congruent	r0.75	block09	0
m01	Fully Congruent	r0.75	block09	1
m01	Incogruent	r0.6	block09	0
m01	Incogruent	r0.6	block09	1
m01	Incogruent	r0.6	block09	1
m01	Fully Congruent	r0.6	block09	1
m01	Fully Congruent	r0.6	block09	1
m01	Incogruent	r0.6	block09	0
m01	Partially Congruent	r0.6	block10	1
m01	Incogruent	r0.75	block10	1
m01	Partially Congruent	r0.75	block10	1
m01	Fully Congruent	r0.75	block10	0
m01	Incogruent	r0.75	block10	0
m01	Fully Congruent	r0.6	block10	1
m01	Fully Congruent	r0.6	block10	1
m01	Fully Congruent	r0.75	block10	1
m01	Fully Congruent	r0.6	block10	1
m01	Fully Congruent	r0.6	block10	1
m01	Incogruent	r0.75	block10	1

m01	Fully Congruent	r0.75	block10	1
m01	Partially Congruent	r0.6	block10	1
m01	Fully Congruent	r0.75	block10	1
m01	Incogruent	r0.75	block10	1
m01	Incogruent	r0.75	block10	0
m01	Fully Congruent	r0.75	block10	1
m01	Incogruent	r0.6	block10	0
m01	Incogruent	r0.75	block10	1
m01	Partially Congruent	r0.75	block10	0
m01	Incogruent	r0.75	block10	1
m01	Incogruent	r0.75	block10	0
m01	Fully Congruent	r0.6	block10	1
m01	Fully Congruent	r0.75	block10	0
m01	Incogruent	r0.6	block10	1
m01	Incogruent	r0.6	block10	1
m01	Incogruent	r0.6	block10	1
m01	Incogruent	r0.6	block10	0
m01	Partially Congruent	r0.6	block10	0
m01	Incogruent	r0.75	block10	0
m01	Incogruent	r0.6	block10	0
m01	Fully Congruent	r0.75	block10	0
m01	Partially Congruent	r0.75	block10	1
m01	Partially Congruent	r0.6	block10	1
m01	Incogruent	r0.75	block10	0
m01	Incogruent	r0.75	block10	1
m01	Fully Congruent	r0.75	block10	1
m01	Partially Congruent	r0.6	block10	0
m01	Incogruent	r0.75	block10	0
m01	Fully Congruent	r0.75	block10	1
m01	Fully Congruent	r0.6	block10	1
m01	Incogruent	r0.6	block10	1
m01	Fully Congruent	r0.75	block10	1
m01	Fully Congruent	r0.6	block10	0
m01	Partially Congruent	r0.75	block10	1
m01	Incogruent	r0.6	block10	0
m01	Partially Congruent	r0.6	block10	1
m01	Incogruent	r0.6	block10	0
m01	Fully Congruent	r0.75	block10	0
m01	Fully Congruent	r0.6	block10	1
